# The citrullinating enzyme PADI4 governs progenitor cell proliferation and translation in developing hair follicles

**DOI:** 10.1126/sciadv.adx4511

**Published:** 2025-09-12

**Authors:** Kim Vikhe Patil, Nil Campamà Sanz, Kylie Hin-Man Mak, Wei Yang, Karl Annusver, Jasson Makkar, David Grommisch, Li Lei, Priyanka Sharma, Ryan R. Driskell, Maria Kasper, Maria Genander

**Affiliations:** ^1^ Department of Cell and Molecular Biology, Karolinska Institutet, Stockholm, Sweden.; ^2^ School of Molecular Biosciences, Washington State University, Pullman, Washington, USA.; ^3^ Institut de Pharmacologie et de Biologie Structurale, IPBS, Université de Toulouse, CNRS, UPS, Toulouse, France.

## Abstract

Posttranslational protein modifications have emerged as a mechanism regulating progenitor cell state transitions during tissue formation. Herein, we exploit the stereotyped hair follicle development to delineate the function of PADI4, an enzyme converting peptidylarginine to citrulline. Single-cell sequencing places *Padi4* in both progenitor and differentiated hair lineage cells and indicates that PADI4 acts to repress transcription during hair follicle development. We establish PADI4 as a negative regulator of proliferation, acting on LEF1-positive hair shaft committed progenitor cells. Mechanistically, PADI4 citrullinates proteins associated with mRNA processing and ribosomal biogenesis, and lack of PADI4 promotes protein synthesis and ribosomal RNA transcription in vivo. Characterizing key translational effectors, we demonstrate that PADI4 citrullinates the translational repressor 4E-BP1 and reveal a cross-talk between PADI4 activity and 4E-BP1 phosphorylation. This work sheds light on how posttranslational modifications affect progenitor cell states and tissue formation.

## Teaser

Citrullination, a posttranslational modification, affects progenitor cell states and tissue formation.

## INTRODUCTION

Tissue development and regeneration require progenitor cell renewal and differentiation. In addition to the established importance of transcriptional effectors and niche-derived signaling cues in directing progenitor behavior (*1*), posttranslational protein modifications (PTMs) are able to diversify the functionality of protein pathway mediators and thus increase signaling complexity. PTMs are chemical modifications of amino acids, representing a nongenetic, additional mechanism for expanding protein function. While extensive work has delineated how histone acetylation and methylation codes act to ensure proper stem cell lineage progression (*2*, *3*) and described how phosphorylation, ubiquitination, and SUMOylation on nonhistone proteins is closely associated with pluripotency and differentiation (*4*–*6*), little is known about how citrullination affects progenitor cells during tissue development.

Hair follicle (HF) development is a powerful model to understand progenitor cell lineage progression. HF morphogenesis is initiated from the epidermis at embryonic day 14 (E14). Sequential mesenchymal-epithelial signaling coordinates HF progenitor cell proliferation, which eventually commits to differentiate into one of the seven lineages required for hair formation (*7*). In hair-producing follicles, the inner root sheath (IRS) (comprising IRS cuticle as well as the Huxley’s and Henle’s layers) establishes a supportive channel for the central hair shaft (HS) [sublayered into medulla (Med), cortex (Cx), and cuticle of the HS] forming lineages. Molecularly, the WNT signaling effector LEF1 not only marks progenitor cells in the lower HF committed to the HS lineages but also is required for proper differentiation of the HS Cx (*8*). In contrast, specification of the IRS-lineage depends on the bone morphogenetic protein (BMP)–target gene and transcription factor GATA3 (*9*, *10*).

Citrullination is the conversion of the positively charged amino acid arginine to neutral citrulline (*11*), thus decreasing the overall protein charge and subsequently affecting conformation, binding affinity and subcellular localization of target proteins. Citrullination is mediated by a family of evolutionary conserved Ca^2+^-dependent peptidylarginine deiminases (PADIs) (*12*), which display distinct expression profiles, target specificity, and subcellular localization (*11*). While proteome-wide detection of citrullination has been challenging due to the small (0.98 Da) increase in peptide mass introduced upon arginine-to-citrulline conversion, recent methodological development has allowed for the identification of >4000 unique citrullinated proteins (*13*), suggesting that citrullination has broad regulatory functions.

Peptidylcitrulline was first described in proteins isolated from growing HFs (*14*–*16*) and was later localized to the IRS and Med, correlating to expression of the citrullinating enzymes PADI1 and PADI3 (*17*). Many PADI1/3 substrates (filaggrins, hornerin, trichohyalin, and S100A3) (*14*, *18*–*22*) are expressed in the growing HF and are functionally required for HS differentiation. Human *PADI3* mutations, affecting both PADI3 folding and enzymatic activity, are linked to uncombable hair syndrome, manifesting as frizzy and fair hair resistant to combing flat (*23*), and central centrifugal cicatricial alopecia, a scarring alopecia found predominantly in women of African ancestry (*24*). In contrast to the established role of PADI3 in remodeling of intermediate filaments, PADI4 is associated with self-renewal and stemness (*25*), involved in NETosis (a program for formation of neutrophil extracellular chromatin traps) (*26*), and is up-regulated in several types of cancer (*27*), suggesting that the mechanism of action of PADI4 is distinct from that of PADI3.

Progenitor cell behavior is closely linked to regulation of translation (*28*, *29*). Both HF and epidermal progenitor cells up-regulate translation when exiting the cell cycle and committing to differentiation (*28*, *30*), a process dependent on canonical mechanistic target of rapamycin (mTOR)/Akt pathway activity and ribosomal biogenesis (*31*). mRNA binding proteins are described targets of PADIs (*32*); however, whether and how citrullinating enzymes affect translation and subsequently progenitor cell behaviors remain to be investigated.

Herein, we use single-cell sequencing of developing HFs and map expression of *Padi4* to both progenitor and committed hair lineage cells. We find that the absence of PADI4 in HFs induces gene expression across HF cell clusters, suggesting that PADI4 acts to negatively affect transcription. In addition, we establish PADI4 as a negative regulator of proliferation, acting on LEF1-positive HS committed progenitor cells. Mechanistically, PADI4 citrullinates proteins associated with mRNA processing and ribosomal biogenesis, and lack of PADI4 promotes protein synthesis and ribosomal RNA (rRNA) transcription in developing HFs. Characterizing key translational effectors, we demonstrate that PADI4 citrullinates the translational repressor 4E-BP1 in cultured progenitor cells and describe a cross-talk between PADI4 and 4E-BP1 phosphorylation. We report that PADI4 contributes to HF development by repressing progenitor cell proliferation and translational activity.

## RESULTS

### PADI4 is expressed in the developing HFs

**Fig. 1.**
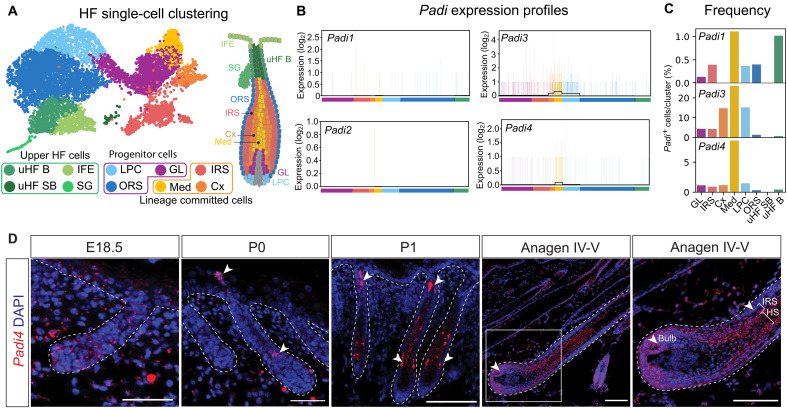
*Padi4* expression in the developing HF. (**A**) UMAP of P1 subpopulations derived from FACS isolation and single-cell RNA sequencing of tdTomato-expressing epithelial cells (K14-Cre:tdTomato). (**B**) Expression levels (individual cell log_2_ count) of *Padi* mRNAs. Clusters are color coded as in (A). (**C**) Frequency of *Padi*-positive cells per HF cluster (percentage). (**D**) In situ hybridization of *Padi4* (red) at multiple developmental time points. Low *Padi4* expression is scattered in the developing HF (E18.5). At P0, *Padi4* expression is detected in the forming Med, as well as in the hair canal (white arrow heads). At P1, *Padi4* mRNA is recorded in differentiated inner HF lineages (white arrows). Ten days postdepilation (adult anagen IV-V), *Padi4* is found in progenitor cells in the lower HF (Mx, matrix), as well as in the inner differentiated HS and IRS lineages. Scale bars, 50 μm (E18.5 and P0) and 100 μm (P1 and adult anagen IV-V). uHF B, upper HF basal; uHF SB, upper HF suprabasal; IFE, interfollicular epidermis; SG, sebaceous gland; LPC, lower proximal cup; ORS, outer root sheath; GL, germinative layer; IRS, inner root sheath; Med, medulla; Cx, cortex.

PADI expression and function is linked to hair formation, both in mice and humans (*23*). To gain a detailed map over PADI expression in developing HFs, we performed single-cell RNA sequencing on fluorescence-activated cell sorting (FACS)–isolated K14-Cre;tdTomato (*33*) reporter–positive epithelial cells at postnatal day 1 (P1). By enzymatically removing the epidermis from the dermis, we were able to enrich for growing HFs extending into the dermis. HF cells were FACS isolated on the basis of their tdTomato expression and transcriptionally profiled. Unbiased cluster analysis revealed that we could faithfully capture cells from all HF lineages: basal and suprabasal cells located in the upper HF (uHF B and uHF SB, respectively; green); proliferative progenitor cells in the outer root sheath (ORS; dark blue), lower proximal cup (LPC; light blue), and germinative layer (GL; purple); lineage committed cells belonging to the IRS (dark orange); and the hair forming lineages themselves (Cx and Med, light orange and yellow) (*34*), in addition to a limited number of cells isolated from the interfollicular epidermis and sebaceous gland (IFE and SG, respectively; green) (Fig. 1A; fig. S1, A and B; and table S1). No significant difference in sequencing coverage was recorded when comparing the total number of counts detected in each cluster, allowing us to directly compare gene expression (fig. S1C).

Probing for *Padi* mRNA expression, we detected *Padi1*, *Padi3*, and *Padi4*, but not *Padi2* or *Padi6*, expression in developing HFs (Fig. 1B and fig. S1, D to F). Previous work placed PADI1 and PADI3 protein in the IRS of human HFs (*17*). Although we detected overall lower levels of *Padi1* compared to those of *Padi3* mRNA, both genes were enriched in the HS lineages (Cx and Med) as well as in the LPC progenitor cluster (Fig. 1C) rather than exclusively marking the IRS. In addition, *Padi1* expression was recorded in the basal cells of the uHF (Fig. 1C). We found *Padi4* to be expressed in a similar pattern as *Padi3*, where the percentage of *Padi4*-positive cells was highest in the differentiated cells of the Med lineage and in the GL and LPC progenitor cell clusters (Fig. 1C), in line with previous reports characterizing adult hair-producing HFs (*34*).

Functionally, PADI1 and PADI3 citrullinates intermediate filaments and their associated proteins required for HS differentiation (*18*, *22*, *35*). Where and how PADI4 impinges on the HF stem cell lineage is, however, unknown. To gain more information of the spatial distribution of *Padi4* during HF development, we used in situ hybridization (RNAscope). At E18.5 (Fig. 1D), *Padi4* mRNA was uniformly distributed at low levels throughout the growing HF and epidermis. In contrast, at the earliest postnatal time point (P0, Fig. 1D), *Padi4* could be detected in the inner HF lineages associated with differentiation of the HS. Curiously, there was also an accumulation of *Padi4* in a cluster of cells corresponding to the formation of the hair canal, a tunnel-like opening that allows for the passage of the growing hair strand as it exits the HF (*36*). At P1 (Fig. 1D), an increase of *Padi4* expression was observed in all differentiating lineages (Cx, Med, and IRS). To gain better cellular resolution, we also probed adult HFs in growth phase. We recorded *Padi4* mRNA in progenitor cells of the lower hair bulb (corresponding to the LPC and GL clusters) as well as in the differentiated lineages of the IRS, Cx, and Med (Fig. 1D). Together, these data suggest that *Padi1*, *Padi3*, and *Padi4* are present throughout the HF stem cell lineage, displaying partly overlapping expression profiles and potentially affecting uncommitted progenitor as well as specified lineage cells*.*

### Loss of PADI4 induces gene expression

**Fig. 2.**
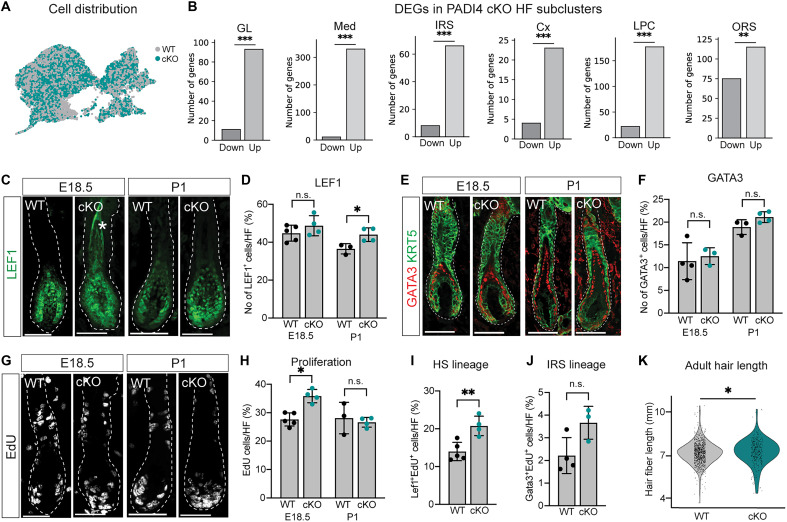
PADI4 negatively regulates HF progenitor cell proliferation. (**A**) UMAP displaying distribution of PADI4 WT and cKO clustered HF cells. Both WT (gray) and cKO (teal) HF cells localize to all clusters. (**B**) Number of differentially expressed genes (DEGs) in all *Padi4*-positive HF clusters, comparing PADI4 WT and cKO. Significantly more genes are up-regulated than silenced in the absence of PADI4. (**C**) Visualization of LEF1-positive HF cells in E18.5 and P1 PADI4 WT and cKO HFs. (**D**) Quantification of the number of LEF1-positive cells per HF at E18.5 and P1. (**E**) Immunofluorescence analysis and (**F**) quantification of GATA3-expressing HF cells in PADI4 WT and cKO at E18.5 and P1. EdU labeling (**G**) and quantification (**H**) reveal an increase in proliferation at E18.5, but not at P1, when comparing PADI4 WT and cKO HFs. Quantification of the percentage of LEF1^+^/EdU^+^ (**I**) or GATA3^+^/EdU^+^ (**J**) double-positive HS- or IRS-committed progenitor cells at E18.5, in PADI4 WT and cKO HFs, respectively. (**K**) Quantification of HS length using adult (second telogen) plucked hairs, comparing PADI4 WT and cKO mice. Each dot represents one HS. (B) DEGs were calculated using the Wilcoxon rank sum method. A gene was considered differentially expressed if adjusted *P* value of <0.05 and log_2_ fold change of >0.5. Statistical comparisons between the number of down- and up-regulated genes were performed using a two-sided binomial test. *P < 0.05, **P < 0.01, ***P < 0.001 (D, F, H, I, and J) Two-sided unpaired Student’s *t* test. (K) Mann-Whitney *U* test, *P < 0.05; each dot represents one HS; *n* = 3 cKO and *n* = 10 WT animals. Wilcoxon rank sum statistical tests were used to compare hair fiber length measurements as normality and equal variance assumptions were not met. (C, E, and G) Scale bars, 50 μm. Data are represented as means ± SD; each dot represents one animal (D, F, and H to J). Asterisk (*) in (C) marks autofluorescence from HS. n.s., not significant.

Given the intriguing expression of *Padi4* in the developing HF, we asked whether PADI4 contributed to HF development. To this end, we crossed the *K14*-Cre;tdTomato line to Padi4^fl/fl^ mice (*33*, *37*), generating a CRE-dependent, skin specific, ablation of *Padi4* [hereafter referred to as PADI4 conditional knockout (cKO)]. P1 PADI4 cKO HF cells were isolated and transcriptionally profiled as previously described (Figs. 1A and 2A). All HF clusters found in PADI4 WT HFs were also retrieved in the absence of PADI4 (fig. S2A), with no obvious differences in cluster abundance (fig. S2B), indicating that the developmental establishment of HF lineages is PADI4 independent.

Because all HF clusters were present in the absence of PADI4, we focused on genes differentially expressed in the six HF clusters where *Padi4* expression was most prominent (GL, Med, IRS, Cx, LPC, and ORS) (Fig. 1B). All six clusters contained significantly more up-regulated than down-regulated genes in the absence of PADI4 (Fig. 2B and table S2) where the progenitor cell clusters (LPC and GL) together with committed Med lineage cells (Med) recorded the highest number of up-regulated genes. PADI4 can translocate to the nucleus, bind chromatin, and act as a transcriptional coregulator (*38*, *39*). Although the observed changes in gene expression are correlative, this uneven distribution of up- and down-regulated DEGs suggests that PADI4 could act as a corepressor during HF development.

To unveil potential cluster-independent, but PADI4-associated, transcriptional changes, we identified genes that were up-regulated in more than one HF cluster (table S2). We found that commonly up-regulated genes included several transcriptional and chromatin regulators, including *Hmga1b*, *Fos*, *Fosb*, and *Egr1* that were up-regulated in all or most of the six clusters (fig. S2C). In addition, we found *Hdac1*, a reported PADI4 interactor (*40*) and a known epigenetic HF lineage effector (*41*), to be up-regulated in the HS lineages (GL-Med-Cx), whereas transcription factors *Jun* and *Id3* were coregulated in the ORS and LPC (fig. S2C). Turning to up-regulated but cluster-specific genes (fig. S2D and table S2), we identified differential expression of HS lineage differentiation markers (*Foxq1*, *Msx1*, *Msx2*, and *Krt35*) in the Med cluster, cell cycle regulators (*PCNA*, *Lef1*, and *Mcm5*) in the LPC, and secreted signaling molecules (*Shh*, *Bmp2*, and *Wnts*) in multiple clusters (fig. S2D). In addition, we found ribosomal genes (*Rrp15*, *Rrp9*, and *Rrs1*) to be up-regulated in the LPC (fig. S2D). These data indicate that PADI4, directly or indirectly, negatively affects expression of common, cluster-independent, as well as lineage-specific transcriptional programs in developing HFs.

### PADI4 restricts proliferation in LEF1-positive progenitor cells

Genes associated with cell cycle progression (*PCNA*, *Lef1*, and *Mcm5*) were up-regulated in the LPC in the absence of PADI4, suggesting that PADI4 could affect HF progenitor cell proliferation (*42*). Consequently, we mapped predicted cell cycle stages of PADI4 WT and cKO cells in all six HF clusters on the basis of their transcriptional states (see Materials and Methods) (fig. S2E). As expected, cells clustering to the GL had the highest predicted percentage of cells in the S and G_2_-M phases, whereas cells in the Cx and Med were less likely to be cycling. PADI4 cKO LPC cells were statistically more likely to be proliferating when compared to their WT counterparts. To understand whether these predicted cell states corresponded to changes in progenitor cell behavior during HF development, we analyzed developing HFs from P1 as well as E18.5, the first time point when we could confidently see hair lineage differentiation markers. We found that the number of LEF1-positive cells was increased at P1, but not E18.5, when comparing PADI4 cKO and WT (Fig. 2, C and D), correlating with the increased *Lef1* expression in the LPC cluster at P1 (fig. S2D). LEF1 is reported to interact with PADI4 in hematopoietic cells (*43*). We therefore overexpressed PADI4 and LEF1 in human embryonic kidney (HEK) 293 cells and performed coimmunoprecipitation, confirming a PADI4-to-LEF1 interaction (fig. S2F). These data suggest that PADI4 could affect the function of LEF1 during HF development and lineage specification.

LEF1 marks undifferentiated progenitor cells committed to the HS (Med and Cx) lineage. We asked whether the increased number of LEF1-positive cells was the result of a skewed lineage specification and, hence, correlated to a decrease in progenitor cells committed to the IRS lineage. Using GATA3 as a marker for IRS-committed progenitor cells, we did not record any statistically significant differences in the number of GATA3-positive cells at either E18.5 or P1, when comparing PADI4 WT and cKO HFs (Fig. 2, E and F). These data suggest that PADI4 acts to expand the LEF1 lineage without negatively affecting the GATA3-lineage specification.

To understand whether the increased number of LEF1-positive cells correlated to increased proliferation during HF development, we quantified the number of cycling, 5-ethynyl-2′-deoxyuridine (EdU)–positive cells per HF. We found that HF proliferation was increased in PADI4 cKO mice at E18.5 (Fig. 2, G and H), but not at later (P1) or earlier (E16.5) developmental time points (Fig. 2, G and H, and fig. S2G) when compared to WT HFs. Focusing on E18.5, we then quantified EdU incorporation in undifferentiated but committed GATA3- and LEF1-positive progenitor cells. We demonstrated a significant increase in proliferation of progenitor cells marked by LEF1, but not GATA3 (Fig. 2, I and J), indicating that PADI4 restricts proliferation of the LEF1-positive progenitor population. Measuring HF length at E18.5 and P1 revealed an enrichment of long (>200 μm) HFs at E18.5 (fig. S2H), a difference that was gone at P1 (fig. S2I). The hairs formed during development are retained into adulthood. We plucked and quantified the length of adult hairs (*44*) and recorded a small, but significant, increase in overall HS length when comparing PADI4 WT and cKO mice (Fig. 2K and fig. S2J). Together, these data indicate that PADI4 negatively regulates proliferation of progenitor cells committed to the HS lineage, affecting the length of the HS.

### PADI4 does not restrict HS differentiation

**Fig. 3.**
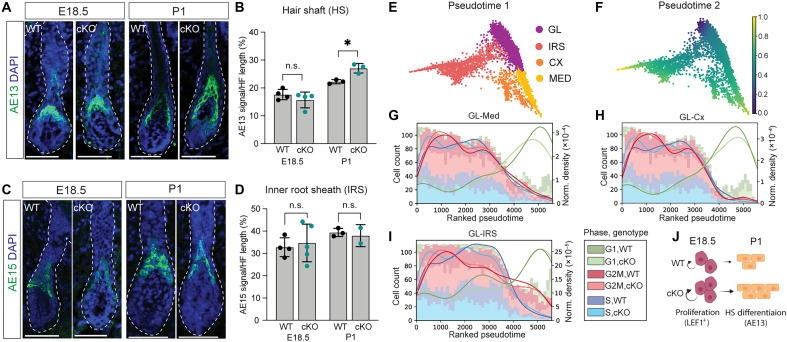
HS differentiation is uncoupled from PADI4. (**A** and **B**) Labeling and quantification of the differentiated HS lineage using AE13 reveals a precocious HS differentiation at P1, but not E18.5, when comparing PADI4 WT and cKO. (**C** and **D**) Labeling and quantification of the differentiated IRS lineage using AE15 at E18.5 and P1, comparing PADI4 WT and cKO. (**E**) Diffusion map (*59*, *61*, *71*) displaying the inner HF clusters with corresponding pseudotime (**F**). Smaller values (blue) represent undifferentiated cell states (GL) and larger values (yellow) mark differentiated cell populations (Med, Cx, and IRS). (**G** to **I**) Histogram and kernel density estimate (KDE) plots show the distribution of cells across ranked pseudotime for the transition from GL to Med (G), Cx (H), and IRS (I). The *x* axis represents the ranked pseudotime, while the *y* axes indicate binned cell count (histogram, left) and normalized density (KDE, right), grouped by cell cycle phase and genotype (PADI4 WT or cKO). (**J**) PADI4 acts to restrict LEF1-positive progenitor cell proliferation at E18.5, resulting in an increase in HS differentiation at P1. Two-sided unpaired Student’s *t* test (B and D); n.s., *P* > 0.05; *P < 0.05. Scale bars, 50 μm (A and C). Data are represented as means ± SD; each dot represents one animal (B and D).

To understand whether the increased proliferation within the LEF1^+^ progenitor population at E18.5 resulted in alterations in HS differentiation, we quantified the expression of the Cx-specific keratin AE13 (K40) at E18.5 and P1 (Fig. 3A). We recorded increased AE13 immunoreactivity in PADI4 cKO at P1, but not E18.5, when compared to that in WT HFs (Fig. 3B). The increased differentiation of the HS lineage did not occur at the expense of the IRS lineage, where no difference in IRS differentiation marker expression (AE15) could be observed at either E18.5 or P1 (Fig. 3, C and D). To uncouple altered differentiation dynamics from increased proliferation, we performed pseudotime analysis (Fig. 3E) (*45*, *46*) of the inner HF cells, defining the GL as the least differentiated starting population (Fig. 3F, dark blue). Pseudotime trajectory indicated that PADI4 WT and cKO GL progenitor cells were equally likely to transition toward a differentiated state, irrespective of cell cycle status or lineage (Med, Cx, or IRS) fate (Fig. 3, G to I). These data suggest that PADI4 affects the HS lineage by restricting progenitor cell proliferation and not differentiation (Fig. 3J).

### PADI4 OE inhibits progenitor cell proliferation

**Fig. 4.**
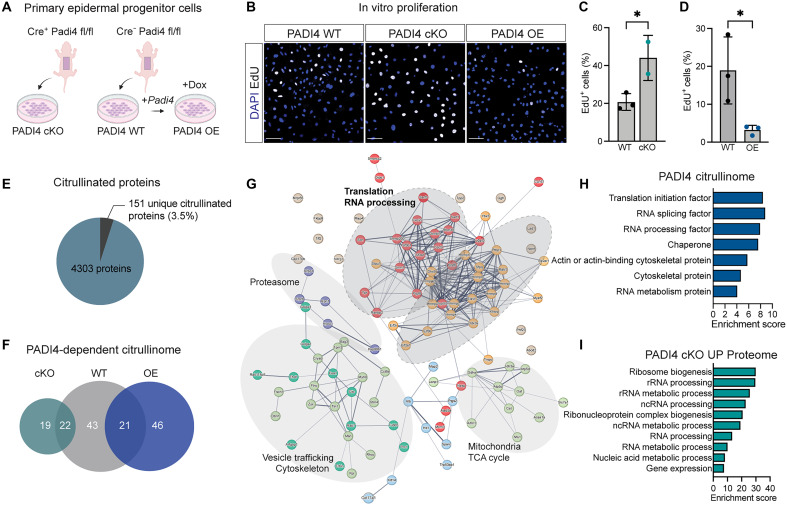
Characterization of the PADI4-dependent citrullinome. (**A**) Schematic illustration of the generation and establishment of primary epidermal progenitor cells from either PADI4 WT or cKO (K14-Cre +/fl or fl/fl) mice at P1. PADI4 WT progenitor cells were transduced with lentivirus coding for a doxycycline-inducible *Padi4* [PADI4 overexpression (OE)]*.* This illustration was created in BioRender (M. Genander, 2025; https://BioRender.com/ijwnlnr). (**B** to **D**) EdU incorporation and quantification using primary PADI4 WT, cKO, and OE progenitor cells. (**E**) Pie chart displaying the number of unique citrullinated proteins (151 cit) out of the whole proteome (4303 tot) as detected across PADI4 WT, cKO, and OE using mass spectrometry analysis. (**F**) Venn diagram recording the number of unique and common citrullinated proteins comparing PADI4 WT, cKO, and OE progenitor cells. (**G**) Protein clustering is performed on the basis of predicted interactions using the STRING platform. Functionally enriched groups are visualized using gray circles and their associated Gene Ontology (GO) terms are displayed. (**H**) GO analysis of PADI4-dependent citrullinated proteins reveal processes associated with RNA processing and translation. (**I**) GO analysis of the proteins up-regulated in PADI4 cKO progenitor cells compared to that in WT. Two-sided unpaired Student’s *t* test (C and D); n.s., *P* > 0.05; *P < 0.05. Scale bars, 50 μm (B). Data are represented as means ± SD; each dot represents one biological replicate (C and D). ncRNA, noncoding RNA.

To verify the effect of PADI4 on progenitor cell proliferation, we established in vitro primary epidermal progenitor cells from both PADI4 cKO and WT littermate controls (see Materials and Methods). Furthermore, we generated progenitor cells where PADI4 expression could be induced using doxycycline [PADI4 overexpression (OE)] (Fig. 4A). Probing the expression of *Padi* family members in vitro, epidermal progenitor cells, analogous to developing HFs, expressed *Padi3* and *Padi4*, but not the additional family members *Padi1*, *Padi2*, and *Padi6* (fig. S3A). Neither PADI4 cKO nor OE progenitor cells displayed any compensatory effect on the expression of *Padi3* in response to ablation or up-regulation of PADI4 (fig. S3, B and C). Assessing proliferative capacity, epidermal progenitor cells lacking PADI4 displayed increased EdU incorporation when compared to PADI4 WT progenitor cells, mimicking the heightened proliferation observed in the PADI4 cKO HFs and validating the in vitro system (Fig. 4, B and C). Conversely, OE of PADI4 resulted in decreased progenitor cell proliferation (Fig. 4, B and D). These results suggest that PADI4 negatively affects progenitor cell proliferation both in vitro and in vivo.

### PADI4 targets proteins regulating RNA dynamics and translation

To gain mechanistic understanding of how PADI4 affects HF development, we characterized PADI4-dependent protein citrullination. Performing high-throughput quantitative proteomics analysis using liquid chromatography–tandem mass spectrometry (LC-MS/MS) on protein lysates isolated from PADI4 cKO, OE, and WT progenitor cells, we identified a total of 4303 proteins out of which 151 unique proteins (3.5% of the proteome) were citrullinated (Fig. 4E and fig. S3D). Citrullinated proteins were found in all progenitor cell samples in comparable numbers (Fig. 4F).

We defined the PADI4-dependent citrullinome as proteins citrullinated in both PADI4 OE and WT, as well as uniquely enriched in PADI4 OE, but absent in PADI4 cKO progenitor cells [110 proteins (43 + 21 + 46)] (Fig. 4F and table S3). We then explored the STRING interactome, which clusters proteins based on interactions, thereby inferring functional enrichments (Fig. 4G). Clustering of the PADI4-dependent citrullinome revealed that PADI4 has a role affecting both translation (targeting translation initiation factors eIF2α, eIF3b, and eIF3i), as well as posttranscriptional regulation (RNA processing) as several RNA helicases (DHX9 and DDX3X), splicing factors (RBM39 and SFPQ), and RNA transporter/trafficking proteins (STAU1 and SNUT1) were found to be citrullinated by PADI4 (Fig. 4G). Gene Ontology (GO) analysis of the PADI4-dependent citrullinome confirmed an enrichment of proteins associated with RNA processing, splicing, translation, and the cytoskeleton (Fig. 4H). These data demonstrate that PADI4 citrullinates proteins central to basic cellular processes such as translation and RNA processing.

Forty-one citrullinated proteins (19 + 22) were detected in PADI4 cKO progenitor cells (Fig. 4F) that, based on the *Padi* expression analysis (fig. S3A), are likely to be mediated by PADI3. GO analysis of the PADI3-dependent (PADI4 cKO) citrullinated proteins revealed an enrichment of proteins associated with apoptosis, cell-to-cell junctions, and differentiation (fig. S3E), indicating that PADI3 and PADI4 differ in their target specificities and have nonoverlapping cellular functions.

### Loss of PADI4 promotes ribosomal biogenesis

The translational machinery is differentially regulated during progenitor lineage progression (*31*). Because our data suggest that PADI4 citrullination affects various aspects of RNA processing and translation, we reasoned that focusing on the proteome, rather than the transcriptome, would be more informative to understand whether and how PADI4 affects progenitor states. By using quantitative tandem mass tag (TMT) labeling, we identified significant changes in progenitor cell proteomes in the absence and presence of PADI4. Comparing up-regulated proteins to up-regulated mRNA (from single-cell data) in PADI4 cKO identified only four common targets [HMGA1b (chromatin binding, also up-regulated in PADI4 cKO HFs; fig. S2C), NASP (regulates nuclear translocation of histones), UBE2s (ubiquitin ligase), and ILRUN (fig. S3F and table S4)], suggesting that PADI4-driven changes in the proteome are not necessarily reflected in the transcriptome.

Focusing on changes in the proteome, we found that progenitor cells lacking PADI4 up-regulated proteins associated with ribosomal biogenesis and rRNA processing (Fig. 4I), confirming that the translational machinery is altered in the absence of PADI4. We then turned to progenitor cells in which PADI4 was overexpressed. However, down-regulated proteins in PADI4 OE progenitor cells were not associated with ribosomal biogenesis but rather linked to the electron transport chain and cytoskeleton (fig. S3G). Furthermore, up-regulated proteins in PADI4 OE progenitors were associated with chromatin remodeling and RNA metabolism (fig. S3H). Given that decitrullinating enzymes are yet to be identified (*13*), citrullination has been suggested to mark proteins for degradation (*22*, *47*). Scrutinizing the proteome to investigate whether enrichment, or absence, of citrullination affected protein levels, we were, however, not able to correlate citrullination to any discernible change in protein abundance (fig. S3I). These data not only indicate that PADI4-mediated citrullination is not a mark for protein degradation but also suggest that forced PADI4 expression is able to expand the number of protein targets and, consequently, the cellular functions affected by citrullination.

### PADI4 negatively affects protein synthesis in progenitor cells

**Fig. 5.**
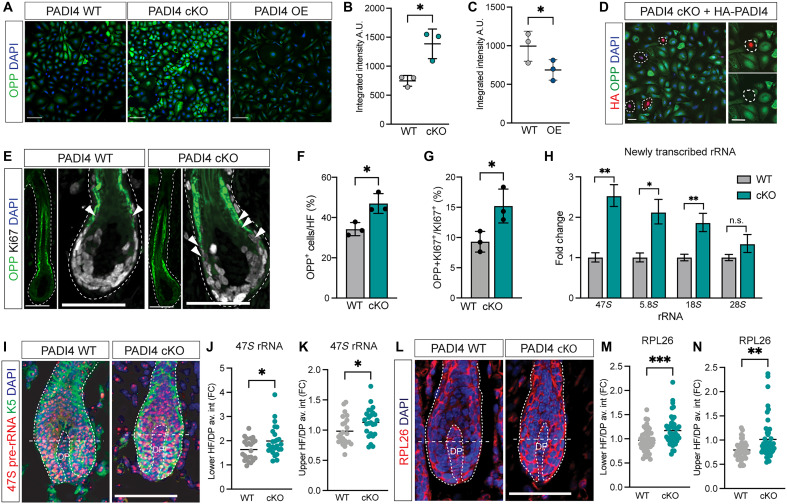
PADI4 inhibits global translational output by targeting ribosomal biogenesis. (**A** to **C**) Visualization and quantification of OPP incorporation intensity in PADI4 WT, cKO, and OE progenitor cells*.* (**D**) Forced expression of HA-tagged PADI4 (red) in PADI4 cKO progenitor cells leads to reduced OPP incorporation (green). (**E** and **F**) In vivo OPP incorporation at P1 is significantly increased when comparing PADI4 WT and cKO HFs. (**G**) In addition, the percentage of Ki67/OPP double-positive cells is higher in PADI4 cKO HFs compared to that in PADI4 WT. (**H**) Nascent rRNA synthesis, labeled using the uridine analog 5-EU for 3 hours. rRNA transcription is significantly increased in PADI4 cKO compared to that in WT progenitor cells. 47*S* represents the pre-rRNA subsequently spliced into 5.8*S*, 18*S*, and 28*S* rRNA. (**I**) In vivo HF global pre-rRNA transcription is visualized using RNAscope in situ hybridization against 47*S*. (**J** and **K**) quantification of HF rRNA synthesis by measuring 47*S* signal intensity in lower (J) or upper (K) HF in PADI4 WT and cKO HFs. Intensity measurements from each HF are normalized against the dermal papilla (DP) of the same HF. (**L**) Localization of the ribosomal protein RPL26 in P1 HFs from PADI4 WT and cKO. (**M** and **N**) Quantification of average signal intensity in lower (M) or upper (N) hair bulb, normalized to the average signal intensity of the DP for each HF. Two-sided unpaired Student’s *t* test (B, C, F to H, J, K, M, and N); n.s., *P* > 0.05; *P < 0.05; **P < 0.01; ***P < 0.001. Scale bars, 50 μm (A, D, E, I, and L). Data are represented as means ± SD; each dot represents a biological replicate (B, C, and H); animal (F and G) or one HF (J to N) was collected from at least three individual animals. A.U., arbitrary units; FC, fold change.

To assess the translation rate in PADI4 WT, cKO, and OE progenitor cells, we administered the puromycin analog *O*-propargyl-puromycin (OPP) in vitro for 30 min. OPP incorporates into newly synthesized peptide chains and can be quantified using Click-iT chemistry. We found that the level of PADI4 correlated to translation rates, where PADI4 cKO displayed increased and PADI4 OE decreased overall protein synthesis rates compared to WT progenitor cells (Fig. 5, A to C). We were able to reduce OPP incorporation to normal levels by reintroducing HA-tagged PADI in PADI4 cKO progenitor cells (Fig. 5D), indicating that PADI4 is directly acting to inhibit overall protein translation.

We then injected PADI4 WT and cKO mice with OPP for 1 hour, allowing us to determine the translational output in HFs in vivo. In line with previous reports (*28*), HF cells with the highest OPP incorporation were committed to lineage differentiation (fig. S4, A and B). Quantification revealed a higher percentage of cells positive for OPP in PADI4 cKO compared to that in WT HFs both at E18.5 and P1 (Fig. 5, E and F, and fig. S4, C and D). There was also a significant increase in progenitor cells coexpressing Ki67 and OPP (Fig. 5G and fig. S4E) in PADI4 cKO compared to those in WT HFs at both time points analyzed. These data demonstrate that, in the absence of PADI4, HF progenitor cells increase their translational output.

### Loss of PADI4 promotes ribosomal biogenesis

Our GO analysis suggested that ribosomal biogenesis was increased in the absence of PADI4 (Fig. 4I). Increased de novo assembly of functional ribosomes requires not only production of ribosomal proteins but also increased rRNA transcription (fig. S4F) (*48*). To address whether rRNA transcription was altered in PADI4 cKO progenitor cells, we labeled actively forming RNA using a 3-hour pulse of the uridine analog 5-ethynyl uridine (5-EU). Quantitative polymerase chain reaction (qPCR) analysis demonstrated that nascent rRNA species (47*S*, 5.8*S*, 18*S*, and 28*S*) were being transcribed at a higher level in PADI4 cKO compared to those in WT progenitor cells (Fig. 5H). Additionally, the total levels of rRNAs were similarly increased in PADI4 cKO compared to those in WT, indicating that the rRNA turnover was not negatively affected by PADI4 (fig. S4G). Analogous to the absence of changes in levels of proteins associated with ribosomal biogenesis in PADI4 OE progenitor cells (fig. S3G), rRNA transcription was not affected upon forced PADI4 expression (fig. S4, H and I), suggesting that the reduced translation observed in PADI4 OE progenitor cells is mediated through additional mechanisms.

To probe whether rRNA transcription was increased in the absence of PADI4 in vivo, we quantified the intensity of the 47*S* pre-rRNA using RNAscope. We recorded a small increase in the mean 47*S* intensity in both the lower (progenitor) and upper (committed to differentiation) hair bulb (Fig. 5, I to K), whereas the 47*S* ratio between lower and upper HF was constant (fig. S4J), suggesting that PADI4 cKO progenitor as well as committed lineage cells display an increase in rRNA production in vivo. We then exploited cultured PADI4 WT and cKO progenitor cells and asked them to differentiate for 24 and 48 hours. OPP administration for 1 hour not only confirmed that translation was increased in progenitor cells (0 hours) in the absence of PADI4 but also demonstrated that the increased translation rate was sustained during differentiation (fig. S4K), suggesting that PADI4 affects translation rates in both progenitor and lineage committed cells.

Revisiting the PADI4 cKO HF single-cell profiling, we identified up-regulation of a handful of ribosomal genes, where *Rpl26* was significantly up-regulated across multiple clusters (table S2 and fig. S4L). Comparing the average RPL26 protein immunoreactivity intensity in PADI4 WT and cKO HFs at P1 demonstrated increased mean RPL26 protein abundance in both the lower and upper hair bulb (Fig. 5, L to N). Although we only record a modest up-regulation of RPL26 and 47*S* pre-mRNA in vivo, these data indicate that absence of PADI4 accelerates the generation of ribosomal proteins as well as transcription of rRNAs needed for generating functional ribosomes and place PADI4 centrally in translational regulation of both progenitor and committed lineage cells.

### Loss of PADI4 affects activation of canonical regulators of translation

**Fig. 6.**
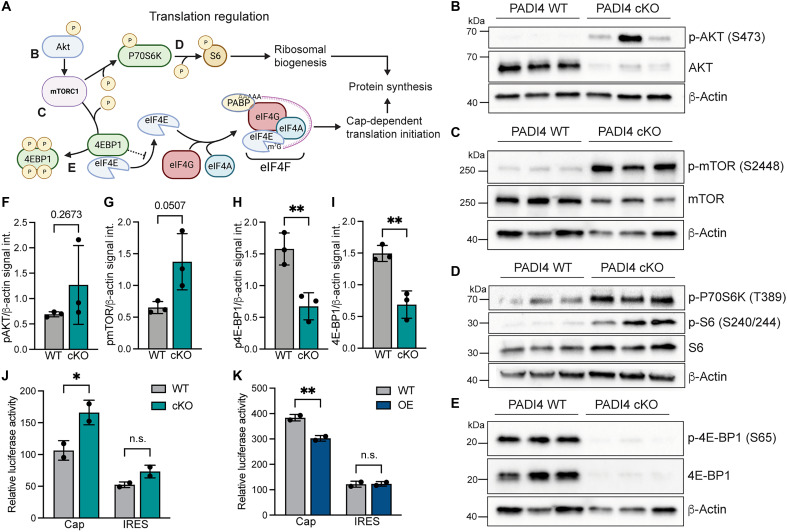
PADI4 rewires the translational machinery in progenitor cells. (**A**) Schematic illustration of the phosphorylation cascade regulating the activity of core translational regulators, including Akt, mTORC1, S6, and 4E-BP1. Together, the targets of mTOR converge to induce protein synthesis. This illustration was created in BioRender (M. Genander, 2025; https://BioRender.com/4jx2r7e). (**B**) Western blot analysis of p-Akt/Akt. Phosphorylation of Akt at serine-473 (p-Akt S473) is increased in PADI4 cKO compared to that in WT progenitor cells, although the total level of Akt is decreased. (**C**) Phosphorylation of mTOR at serine-2448 (p-mTOR S2448) is increased in PADI4 cKO compared to that in WT, indicating p-Akt activity on mTOR, whereas total levels of mTOR are unperturbed. (**D**) Phosphorylation of P70S6K at threonine 389 (p-P70S6K T389) is increased in PADI4 cKO, as is phosphorylation of its target S6 (p-S6 S240/244). (**E**) 4E-BP1 displays both less phosphorylated and total protein levels in PADI4 cKO compared to those in WT progenitor cells. (**F**) β-Actin was used as loading control, and each replicate represents an individual progenitor cell line (B to E). (F) Quantification of p-Akt protein levels normalized to loading control. (**G**) Quantification of p-mTOR protein levels normalized to loading control. (**H**) Quantification of p-4E-BP1 protein levels normalized to loading control. (**I**) Quantification of 4E-BP1 protein levels normalized to loading control. (**J** and **K**) Luciferase assay using reporter constructs for cap- or IRES-dependent translational initiation, comparing PADI4 WT and cKO (J) or PADI4 WT and OE (K) progenitor cells. Two-sided unpaired Student’s *t* test (F to I and G to H); n.s., *P* > 0.05; *P < 0.05; **P < 0.01. Data are represented as means ± SD; *n* = 3 for each condition (F to I). (J and K) *n* = 2; data represent one of the two experiments. The same β-actin loading control is displayed twice in (C) and (E).

Given PADI4’s effect on translation (Fig. 5), we probed the translation initiation machinery focusing on the canonical PI3K/Akt/mTOR pathways that activate and regulate translation (Fig. 6A). Although the total levels of Akt was reduced in PADI4 cKO compared to WT progenitor cells, phosphorylation of Akt (p-Akt S473) was markedly increased, indicating Akt pathway activation (Fig. 6, B and F). Phosphorylation of mTOR (p-mTOR S2448) [downstream of Akt and a reporter of mTOR complex 1 (mTORC1) activity] was also increased in the absence of PADI4 (Fig. 6, C and G). Activated mTOR promotes protein synthesis by phosphorylating P70 S6 kinase 1 (P70S6K). Activated by phosphorylation, P70S6K, in turn, phosphorylates ribosomal protein S6, a component of the 40*S* subunit of the ribosome. Both P70S6K and S6 were found to be distinctly more phosphorylated in the PADI4 cKO compared to those in WT (Fig. 6D), highlighting the involvement of this canonical pathway in mediating increased translational output. Unexpectedly, the phosphorylation and total levels of either of the Akt/mTOR signaling constituents were unaltered when PADI4 was overexpressed (fig. S5, A to C), indicating that the reduced translation rates observed in PADI4 OE progenitor cells are mediated through other mechanisms.

### PADI4 cKO progenitors maintain cap-dependent translation in the absence of 4E-BP1

Activated mTOR phosphorylates eukaryotic initiation factor 4E-binding proteins (4E-BPs), allowing the formation of the eIF4F complex and initiation of 5′ cap-dependent translation (Fig. 6A). However, in the PADI4 cKO, both total protein levels and phosphorylated levels of 4E-BP1 were down-regulated when compared to those in WT progenitor cells (Fig. 6, E, H, and I, and fig. S5D), in stark contrast to the conventional model by which mTOR activates protein synthesis. Moreover, total levels, as well as the phosphorylation, of eIF4E were also down-regulated (fig. S5E). Collectively, these data suggest that, in the absence of PADI4, Akt/mTOR signaling is active but mediates translation in an unconventional manner, circumventing the need for 4E-BP1.

Considering the low expression and phosphorylation levels of 4E-BP1 in vitro, we asked whether the increase in translational output could be caused by activation of alternative translation initiation pathways. One such alternative is the utilization of internal ribosomal entry sites (IRESs), an ordered structure on certain mRNAs that allows for the start of translation without the need of the cap (*49*). However, measuring cap- and IRES-dependent translation initiation rates using luciferase reporter constructs (*50*) revealed that the observed changes in OPP incorporation (Fig. 5, A to C) were mediated through cap-dependent translation, which was increased in the absence of PADI4 and silenced when PADI4 was overexpressed (Fig. 6, J and K). The engagement of cap-dependent translation was further corroborated by the absence of phosphorylated form of eIF2α (p-eIF2α), an indicator of activated integrated stress response, another type of alternative, cap-independent, translation (fig. S5F). These data collectively suggest that PADI4 expression correlates to alterations in cap-dependent translation initiation rates.

### Translational regulation in developing HFs

Aiming to understand the involvement of Akt signaling in the increased translation and ribosomal biogenesis that we observed in vivo, we mapped p-Akt expression in developing HFs. We found p-Akt immunoreactivity to be enriched in the uHF at both E18.5 and P1, and not primarily in progenitor cells located at the bottom of the HF (fig. S5, G and I). Although quantification of the number of p-Akt– and OPP-marked cells revealed a significant increase in cell expressing both markers at E18.5, but not P1, HFs (fig. S5, H and J), we conclude that altered Akt signaling is likely not responsible for the observed increase in HF progenitor cell translation.

We then characterized p-4E-BP1 expression. In contrast to progenitor cells expanding in vitro, we recorded no difference in the number of p-4E-BP1–expressing HF cells when comparing PADI4 cKO and WT HFs as would have been expected on the basis of our in vitro data (fig. S5, K and L). Rather, we recorded an increase in the number of OPP-positive cells also expressing p-4E-BP1 (fig. S5M) in line with the canonical function of phosphorylated 4E-BP1. Together, these data suggest that regulation of translation is cell type specific and that not all aspects of PADI4-mediated translational regulation that we describe in vitro are mirrored in developing HFs in vivo.

### PADI4 binds and citrullinates 4E-BP1

**Fig. 7.**
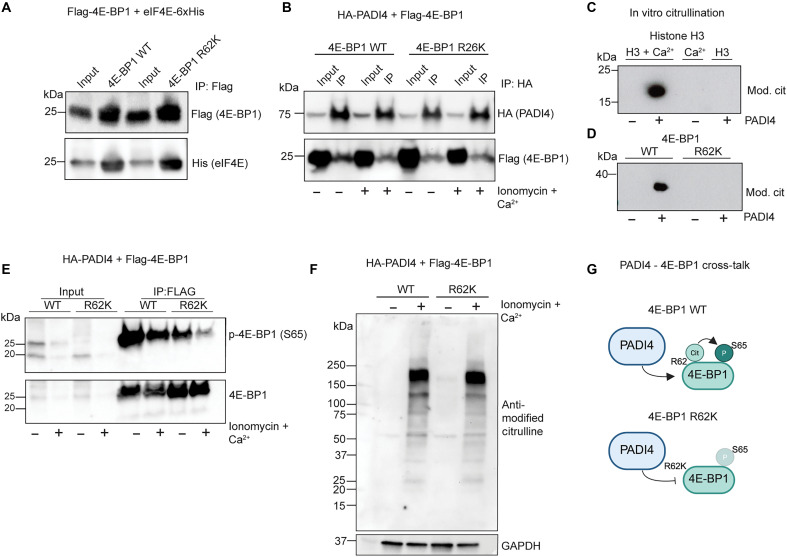
PADI4 binds and citrullinates 4E-BP1. (**A**) Flag-tagged 4E-BP1 WT and 4E-BP1 R62K are both able to coprecipitate His-eIF4E, suggesting that the R62K mutation is not negatively affecting 4E-BP1 protein function. (**B**) Coexpression of HA-PADI4 and Flag-4E-BP1 followed by immunoprecipitation using an anti-Flag antibody demonstrates that PADI4 (top) can interact with 4E-BP1 WT and 4E-BP1 R62K (bottom) under both baseline (−ionomycin and Ca^2+^) and high PADI4 enzyme activity (+ionomycin and Ca2^+^). (**C**) In vitro citrullination of histone H3 using purified recombinant PADI4. PADI4 readily citrullinates histone H3 in the presence of Ca^2+^. (**D**) In vitro citrullination assay using 4E-BP1 WT (left) and R62K (right) recombinant protein incubated with PADI4. PADI4 citrullinates 4E-BP1 WT, but not the R62K mutant. (**E**) Coexpression of HA-PADI4 and Flag-4E-BP1 WT or Flag-4E-BP1 R62K followed by immunoprecipitation using anti-Flag antibody. Phosphorylation of 4E-BP1 at S65 is reduced when R62 is mutated and most pronounced when PADI4 enzyme activity is enhanced using ionomycin and Ca^2+^ (top). Total levels of 4E-BP1 are not altered by the R62K mutation or PADI4 enzyme activity (bottom). (**F**). Verification of PADI4 enzyme activity after ionomycin and Ca^2+^ treatment visualizing citrullinated proteins in total lysate of cell cotransfected with PADI4 and 4E-BP1 WT or 4E-BP1 R62K. Glyceraldehyde-3-phosphate dehydrogenase (GAPDH) was used as a loading control. (**G**) Illustration describing suggested cross-talk of PADI4-mediated 4E-BP1 citrullination and phosphorylation. This illustration was created in BioRender (M. Genander, 2025; https://BioRender.com/h26g2xm).

Unphosphorylated 4E-BP1 acts as a key negative regulator of translation initiation by sequestration of eIF4E. Phosphorylated 4E-BP1 will, however, release eIF4E and enable translation initiation. Citrullination and phosphorylation can function interdependently when occurring on neighboring amino acid residues (*51*), raising the possibility that PADI4 could act directly on 4E-BP1 in cultured progenitor cells. Exploring previously published work reporting >4000 citrullinated proteins in activated human neutrophils (*13*), we identified three arginine residues in 4E-BP1 (R63, R73, and R99) reported to be citrullinated by PADI4, one of which is conserved in the mouse 4E-BP1 (human R63, corresponding to mouse R62) (fig. S6, A and B). We expressed Flag-tagged WT or mutated (R62K) 4E-BP1 in HEK293 cells and found that both versions could coprecipitate eiF4E-6xHis (Fig. 7A). 4E-BP1 normally undergoes proteasomal degradation. Treating PADI4 cKO progenitor cells with MG132, a proteasomal inhibitor, for 24 hours resulted in enrichment of both WT and R62K 4E-BP1 protein (fig. S6C), indicating that the protein stability of the R62K mutant is largely normal.

We then coexpressed Flag-tagged WT or R62K mutated 4E-BP1 with HA-tagged PADI4 (Fig. 7B). Immunoprecipitating PADI4 (using anti-HA antibody-conjugated beads), we detected interaction with both WT and mutated 4E-BP1 (anti-Flag) normally, as well as after treatment with ionomycin and Ca^2+^ to stimulate PADI4 enzymatic activity (Fig. 7B). These data suggest that a small pool of available 4E-BP1 can interact with PADI4.

To understand whether PADI4 also can citrullinate 4E-BP1, we performed in vitro citrullination experiments using recombinant purified PADI4, 4E-BP1 WT, and 4E-BP1 R62K (Fig. 7, C and D). Commercially available purified histone H3 was used to verify PADI4 enzymatic activity. As expected, we recorded induction of prominent H3 citrullination when H3 was incubated together with PADI4 in the presence, but not the absence, of Ca^2+^ (Fig. 7C) (*13*). We then turned to 4E-BP1 and recorded citrullination of the WT, but not R62K mutated, 4E-BP1 protein, when incubated together with PADI4 (Fig. 7D). These data demonstrate that 4E-BP1 R62 can be citrullinated by PADI4 in vitro.

To probe a potential interplay between 4E-BP1 R62 citrullination and S65 phosphorylation, we again overexpressed WT and R62K mutated 4E-BP1 in the presence of PADI4. When probing for S65 phosphorylated 4E-BP1, we find that the 4E-BP1 R62 mutant is less phosphorylated both under baseline PADI4 activity (−ionomycin and Ca2^+^) and upon PADI4 hyperactivation (+ionomycin and Ca^2+^) (Fig. 7E). We confirmed an increased PADI4 enzymatic activity after ionomycin by visualizing citrullinated proteins on Western blot (anti-modified citrulline) (Fig. 7F). These data indicate that PADI4 can interact directly with the translational inhibitor 4E-BP1 and that 4E-BP1 R62 mutation negatively affects the 4E-BP1 S65 phosphorylation (Fig. 7G).

To understand whether cross-talk between citrullination and phosphorylation could be a common mechanism for fine-tuning protein production, we revisited our mass spectrometry data (table S3). However, we were not able to detect citrullination of the canonical translational mediators whose phosphorylation status we previously determined by Western blot (Fig. 6). Turning to a more comprehensive citrullination atlas, however, revealed citrullination marks in both Akt, S6 and P70S6K (*13*). Intriguingly, S6 displayed 22 citrullinated residues,several of which were enriched in the C-terminal protein region that undergoes extensive phosphorylation upon S6 activation (fig. S6D), suggesting that citrullination may play a more important role in regulating translation than previously appreciated.

## DISCUSSION

Protein posttranslational modifications are undisputedly important for the regulation of protein function and, in extension, progenitor cell states and tissue development. Although the PTM landscape has largely been painted with a limited number of contributors, the involvement of less established PTMs is steadily being uncovered in a variety of settings (*52*, *53*). Herein, we describe the contribution of PADI4-mediated citrullination to the HF progenitor lineage.

Exploring the developing HF, we show that *Padi4* is expressed by progenitor as well as committed HF lineage cells (Fig. 1). We observe that genetic ablation of *Padi4* results in a transient proliferative burst of LEF1-positive progenitor cells, bolstering HS differentiation (Fig. 2). These data suggest that PADI4 is important in restricting the LEF1-dependent HS progenitor pool size. Intriguingly, we find increased *Lef1* transcription in vivo and confirm PADI4-LEF1 protein interaction in vitro (*43*), indicating the multiple avenues that PADI4 could act on the LEF1 lineage. Casting a wider regulatory net, BMP signaling, through pSMAD1/5, binds *Padi4* chromatin in adult HFs (*10*), and increased BMP signaling in HS progenitor cells is linked to reduced HS formation (*54*), suggesting a molecular mechanism where BMP-driven *Padi4* expression acts to curb the effects of WNT-LEF1 activity in the HS lineage.

Using cultured progenitor cells to map the citrullinome, we show that PADI4-mediated citrullination is enriched on proteins associated with RNA processing, splicing and translation (Fig. 4). In addition, we demonstrate that the absence of PADI4 also leads to up-regulation of rRNA transcription, both in cultured progenitor cells and in HFs (Fig. 5), resulting in increased availability of rate limiting rRNA species required for translation. These data suggest that PADI4 acts to affect translation through two distinct mechanisms: directly citrullinating protein targets and, by mechanisms still unknown, modulating rRNA transcription. Collectively, these data represent a not previously described function of PADI4, traditionally associated with histone citrullination (*25*).

It is a recurring theme that few of the pathways and proteins increased or decreased in the absence of PADI4 are reversed when PADI4 expression is forced (Fig. 4 and fig. S3). Despite this, PADI4 cKO and OE progenitor cells display opposite phenotypes with regard to both proliferation and translation (Figs. 2, 4, and 5). Reconciling these findings would imply that PADI4-mediated citrullination has the potential to target a large collection of proteins involved in many cellular processes, which, collectively, through different molecular mechanisms, modulate specific aspects of cell behavior. In short, increasing PADI levels or enzymatic activity will not necessarily only positively affect the citrullination stoichiometry for a specific protein but rather expand the number of distinct protein species available for citrullination.

When investigating the molecular pathways that assert a major degree of control on translation, we were most puzzled that the absence of PADI4 resulted in a distinct reduction of 4E-BP1 expression in general and S65 phosphorylation in particular (Fig. 6). An increase in 5′ cap-dependent translation is conventionally mediated through 4E-BP1 phosphorylation, which, in turn, releases eIF4E (Fig. 6). However, our work demonstrates that loss of PADI4 leads to a reduction of the 4E-BP1 pool, and, as a result, up-regulation of translation follows. In other words, loss of PADI4 circumvents the need for phosphorylation of 4E-BP1 to initiate cap-dependent translation. The fact that PADI4 citrullinates 4E-BP1 and that R62-mutated 4E-BP1 is hypo-phosphorylated suggests not only that 4E-BP1 is a target for PADI4 normally but also that phosphorylation and citrullination of 4E-BP1 are interdependent (Fig. 7). We were not able to record reduced 4E-BP1 phosphorylation in HFs, suggesting that the activity and preferred modus operandi of PADI enzymes is highly dependent on the cellular context.

The work presented herein entails the first description of *Padi4* expression and function in developing HFs. It is interesting to note how *Padi3* and *Padi4* expression seems to go hand in hand in progenitor cell clusters as well as the HS lineages. Why the requirement for citrullination would be more enunciated in these specific cell states is relevant to consider, particularly in the light of citrullination as a nonreversible posttranslational modification. PADI3 is known to target cytoplasmic intermediate filaments in differentiated HF cells, but in vivo HF progenitor cell targets have not been described. Despite the abundance of literature placing PADI4 in the context of transcriptional and epigenetic regulation, our data clearly demonstrate that PADI4 also targets proteins that can localize to the cytoplasm, suggesting that the collective contribution of PADI3 and PADI4 to HF progenitor cell states could be substantial. Progenitor cells in the HF are short-lived by nature, only surviving until the end of the hair cycle. Future work will determine whether combined PADI3- and PADI4-mediated citrullination is an essential molecular mechanism required to allow this transient HF progenitor population to properly balance proliferation, lineage commitment, and differentiation within a limited space and time.

Herein, we report that *Padi4* is expressed in developing HFs, negatively regulating proliferation of progenitor cells committed to the HS lineage. Furthermore, we demonstrate that PADI4 citrullinates proteins associated with regulation of translation and that loss of PADI4 increases rRNA transcription. Last, PADI4 binds and citrullinates the translational inhibitor 4E-BP1. This work shows how PADI4 and citrullination is integrated in regulating progenitor cell behavior in vivo, calling into view the importance of citrullination in tissue formation and regeneration.

### Limitations of the study

The impact of PADI4 on specific HF cell populations is extrapolated from mRNA expression profiles, and not from in vivo visualization of PADI4 protein and/or citrullination of established PADI4 targets, such as citrullinated histone H3. Depending on PADI4 protein stability, it is possible that mRNA and protein/enzymatic activity is not fully overlapping. Previous work has shown that citrullination is context dependent. It is, therefore, likely that the PADI4 protein targets that we identify in cultured epidermal progenitor cells are not identical with the in vivo HF targets.

## MATERIALS AND METHODS

### Mouse husbandry

The mouse lines used in this study were *Krt14*-Cre (*33*) crossed to the B6.Cg-*Padi4^tm1.2Kmow/J^* [Jackson Laboratory, no. 026708 (*Padi4f^l/fl^*)] and tdTomato Ai14 (Jackson Laboratory, no. 007914) line. Animals were housed in pathogen-free conditions according to the recommendations of the Federation of European Laboratory Animal Science Association. All experimental procedures on animals were performed following the European directive 2010/63/EU, local Swedish directive L150/SJVFS/2019:9, Saknr L150, and Karolinska Institutet complementary guidelines for procurement and use of laboratory animals, Dnr. 1937/03-640. The procedures described were approved by the regional committee for ethical experiments on laboratory animals in Sweden (Stockholms Norra Djurförsöksetiska nämnd), ethical permit numbers N116/16 and 14051-2019.

### Genotyping

Ear or tail biopsies were lysed in DirectPCR lysis reagent (BioSite) in the presence of proteinase K (0.1 mg/ml; Thermo Fisher Scientific) at 55°C overnight. The reaction was heat inactivated at 85°C for 45 min. Taq polymerase (5 U/μl; final concentration, 0.05 U/μl) was used for gene amplification in polymerase chain reaction (PCR) buffer supplemented with 0.08 to 0.16 mM MgCl_2_, 0.2 mM deoxynucleotide triphosphates (dNTPs; all Invitrogen), and 0.4 μM primers. PCR products were analyzed on a 1.5% agarose gel with GelRed nuclear stain (Biotium).

### Hair collection and analysis

Hair fiber samples were collected from 8-week-old mice by gently plucking hairs from the lower back using tweezers. Hair fibers were extracted from digital images and quantified as previously described (*44*). The image analysis pipeline was slightly modified to accommodate SVS input files directly without need for conversion.

### FACS isolation of HF populations

At P1, mouse back skins were dissected and put in 1:1 1× dispase:phosphate-buffered saline (PBS) [from 5× (10 mg/ml) dispase stock] at 4°C overnight with the dermal side facing the enzymatic solution. After incubation, the epidermis and dermis were mechanically separated using fine forceps. The dermal fraction, containing the growing HFs, was incubated in Hanks’ balanced salt solution (Thermo Fisher Scientific; Gibco) with 20% collagenase (in PBS; Sigma-Aldrich) at 37°C for 1 hour. After incubation, the dermal/HF fraction was scraped off using a scalpel and collected by centrifugation at 300*g* for 10 min, after which the pellet was resuspended in trypsin (Thermo Fisher Scientific; Gibco) and incubated for 15 min at 37°C. Cell suspensions were then filtered using both 70- and 40-mm filters, washed twice in PBS, and pelleted by centrifugation for 5 min at 300*g*. 4′,6-Diamidino-2-phenylindole (DAPI) was used to exclude dead cells. Epithelial cells were tdTOMATO positive. Using a fluorescence-activated cell sorter (BD FACS AriaII), ~100,000 sorted tdTOMATO-positive cells from each animal were collected in E-low medium without serum for subsequent single-cell library preparation.

### Single-cell RNA sequencing library generation

Single-cell libraries were prepared using the Chromium Next GEM Single Cell 3′ v3.1 Reagent Kits (10x Genomics, PN-1000269) according to the manufacturer’s protocol (CG000315 Rev. E). Single-cell suspensions were adjusted to achieve a target recovery of ~10,000 cells. Cells were loaded onto a Chromium Next GEM Chip G along with barcoded gel beads, master mix reagents, and partitioning oil to create Gel Beads-in-Emulsion (GEMs) using the Chromium Controller. Each GEM contained a unique 10x barcode and unique molecular identifier (UMI) linked to mRNA transcripts through reverse transcription. GEMs were incubated to perform reverse transcription, after which they were broken, and cDNA was recovered using Dynabeads with Dynabead cleanup buffer (no. 2000048). First-strand cDNA was amplified by PCR to generate sufficient material for library construction. The amplified cDNA was subjected to enzymatic fragmentation, end-repair, and A-tailing to optimize fragment size. Adapters were ligated, and sample indices were introduced via sample index PCR. Cleanup steps were performed throughout the process to purify and size select cDNA and libraries. Library quality and concentration were assessed using an Agilent Bioanalyzer with high-sensitivity DNA reagents. The final libraries consisted of paired-end constructs with dual indices. Libraries were pooled and sequenced to obtain paired-end reads, with read 1 capturing the 10x barcode and UMI information and read 2 covering the cDNA insert.

### Single-cell analysis

Preprocessing of the sequencing reads into count matrices was done with CellRanger (*55*). Raw count matrices were filtered using CellBender with parameters --expected-cells = 10,000 and --total-droplets-included = 15,000. The filtered counts were processed downstream using Scanpy (*56*). Count matrices of WT and Padi4 cKO were combined and cells following either of these criteria were filtered out: (i) >20% of mitochondrial counts, (ii) <5 or >99 percentile of ribosomal counts, (iii) more than 1% of hemoglobin, (iv) <2000 or >60,000 total counts, or (v) <1000 or >8000 number of genes with positive counts in the cell. Genes were filtered to keep only those (i) expressed in at least five cells and (ii) with at least five counts. Counts were log normalized, and the top 1000 highly variable genes were selected for downstream analysis. Cell cycle phase was predicted using Scanpy’s score_genes_cell_cycle function using previously published cell cycle marker genes (*57*). After performing principal components analysis, the effect of cell cycle phase was corrected using harmony (*58*). The resulting filtered and processed counts were spatially embedded using UMAP (*59*), and clustering was performed using the Leiden algorithm (*60*). Clusters were annotated on the basis of differentially expressed genes found with a Wilcoxon and *t* test. For downstream analysis, fibroblasts, melanocytes, and clusters with mixed signature were removed. Diffusion maps of the inner HF clusters were computed using Scanpy’s implementation (*61*), and its components were selected on the basis of sufficient distinction of the three lineages. Pseudotime analysis was performed using diffusion pseudotime (*45*). Pseudotime values were ordered (ranked pseudotime) and/or binned (50 bins) to visualize cell distributions along differentiation trajectories. Note that tracks plots may result in visualization discrepancies due to rendering a high number of individual cells as thin bars. 

### Primary epidermal progenitor cell isolation and expansion

To establish primary epithelial progenitor cell lines, cells from back skin of P0 *K14*-Cre:*Padi4f^l/fl^* mice were isolated and maintained as previously described (*62*, *63*). Cell lines were established using mitomycin C (Sigma-Aldrich)–inactivated 3T3-J2 feeder cells for cultivation and kept in low–calcium concentration (63.53 μM CaCl_2_) keratinocyte medium {E-low medium [75% high-glucose Dulbecco’s modified Eagle’s medium (DMEM), no Ca^2+^, and no glutamine; 25% Ham’s F12 nutrient mix; 1% penicillin-streptomycin; 8 mM l-glutamine; and 0.03% sodium bicarbonate (all from Thermo Fisher Scientific)], supplemented with 15% calcium free fetal bovine serum (FBS; HyClone) containing hydrocortisone (0.45 μg/ml; Merck), 0.1125 nM cholera toxin (Sigma-Aldrich), transferrin (50 μg/ml; Sigma-Aldrich), insulin (50 μg/ml; Sigma-Aldrich), and 0.02 nM 3,3′,5-triiodo-l-thyronine (Sigma-Aldrich)}. Ear biopsies were collected and processed for genotyping to assess which individual mouse was Cre positive and negative for cKO and WT cell lines, respectively. Of a total of 15 P0 mouse skins plated, 13 were able to establish themselves as cell lines. The epithelial cells were taken off feeders after three passages.

### OE of PADI4 in primary epidermal progenitor cells (PADI4 OE)

N-terminal FLAG-tagged mouse *Padi4* was cloned into the pCW57.1 vector backbone (Addgene, plasmid no. 41393) containing a hygromycin selection cassette. Epidermal progenitor cells expressing protein coding sequences for PADI4 were generated by lentiviral infection followed by hygromycin (200 μg/ml) selection. PADI4 expression was induced by doxycycline (1 μg/ml) for 3 days. Primary epidermal progenitors were within 20 passages. Lentiviral particles were produced in 293FT cells (System Biosciences) expanded in DMEM with 10% FBS, as previously described (*64*), using pMD2/pPAX packaging plasmids (pMD2.G, Addgene, plasmid no. 12259; psPAX2, Addgene, plasmid no. 12260). Epidermal progenitors were infected by spinoculation (1100*g* for 30 min at 37°C) in the presence of hexadimethrine bromide (40 μg/ml).

### RNAscope in situ hybridization

RNA in situ hybridization was conducted using a RNAscope Multiplex Fluorescent V2 kit from Advanced Cell Diagnostics (ACD), according to the manufacturer’s instruction for formaldehyde-fixed paraffin-embedded (FFPE) samples. In brief, back skin of E18.5, P0, and P1 pups were collected within 5 min of animal sacrifice and fixed overnight in 4% formaldehyde (Sigma-Aldrich) at 4°C. Samples were dehydrated and paraffinized, followed by microtome sectioning at 10 µm, and mounted on SuperFrost Plus glass slides. To commence in situ hybridization, FFPE slides were incubated at 60°C for 1 hour, followed by deparaffinization with two 5-min washes of xylene and two 2-min washes of 100% ethanol. The slides were then dried for 5 min at 60°C, followed by incubation with hydrogen peroxide for 10 min at room temperature. The samples then underwent antigen retrieval by being heated in antigen retrieval buffer (tris-citrate, pH 9) at ~95°C in a steamer for 10 min, after which they were treated with protease III at 40°C in the oven for 30 min, followed by incubation with the RNAscope probe for 2 hours at 40°C. After amplification with amplification reagent 1 (AMP 1), AMP 2, and AMP 3, the channel was developed by incubating horseradish peroxidase (HRP), fluorophore, and HRP blocker sequentially. The samples were counterstained with DAPI. Images were captured using a Zeiss Axioplan microscope and processed with Zen blue software. Probes used were directed again *Padi4* (ACD 467591-C2) and 47*S* (ACD 417341).

### OPP and EdU labeling

For the in vivo measurements of protein synthesis, E18.5 embryos were labeled with OPP (MedChemExpress) by intraperitoneal injection of the mother with 50 mg/kg (10 M for each kg of body weight) OPP in PBS 1 hour before sacrifice, whereas P1 pups were injected subcutaneously under the scalp 1 hour before sacrifice. To label cell proliferation in vivo, pregnant dams were intraperitoneally injected with EdU (Thermo Fisher Scientific) in PBS at a concentration of 4 mM for 2 hours before sacrifice, whereas P1 pups were injected subcutaneously under the scalp, also for 2 hours. Cells grown on glass chamber slides were treated with either 20 mM OPP for 30 min or 20 mM EdU for 2 hours before being washed with PBS and fixed. OPP was visualized using the Click-iT Plus OPP Kit (Thermo Fisher Scientific, no. C10456) and EdU using Click-iT EdU Cell Proliferation Kit (Thermo Fisher Scientific, no. C10337).

### Immunofluorescence

Back skins were dissected and embedded in OCT (Tissue-Tek), frozen and cryosectioned at 10 to 13 µm at −20°C, and mounted on room temperature SuperFrost Plus glass slides and left to dry in the cryostat for 1 hour. Samples were fixed for 10 min using 4% formaldehyde (Sigma-Aldrich) in PBS, except sections stained with anti-mouse primary antibodies, where no fixation was performed. Sections were then blocked and permeabilized with 0.3% Triton X-100 (Sigma-Aldrich), 2.5% normal donkey serum (Jackson ImmunoResearch), 2.5% normal goat serum (Jackson ImmunoResearch), and 0.5% bovine albumin serum (Sigma-Aldrich), in PBS for 1 hour at room temperature. Samples were incubated overnight at 4°C with primary antibodies diluted in blocking buffer, followed by incubation with Alexa Fluor–conjugated secondary antibodies (Jackson ImmunoResearch) for 1 hour at room temperature. DAPI was used for nuclear stain, and images were captured using a Zeiss Axioplan microscope and processed with Zen blue software.

### Quantification HF marker expression

At least three cross sections from three to five individual mice were quantified for each HF marker. All specimens were subjected to the same staining procedure, and image acquisition was performed with the same microscope settings. Only HFs that were sectioned along the HF axis were included in the analysis. ImageJ was used for quantifications after the unspecific background signal was removed, and the same arbitrary threshold was applied to all images. Marker-positive and marker-negative cells were manually counted. The ImageJ measure function was used to determine the total length of HFs (from base to epidermis), as well as the relative length of AE13 and AE15. QuPath was used to identify cells based on DAPI, after which 47*S* pre-rRNA intensity or RPL26 protein expression was measured in upper and lower hair bulb and normalized to the expression in the dermal papilla. For each HF quantified using QuPath, 60 to 150 cells were included in the analysis.

### Antibodies used for IF

K5 (BioLegend, 905901; 1:500), LEF1 (Cell Signaling Technology, 2230T; clone C12A5; 1:1000), GATA3 (Santa Cruz Biotechnology, sc-268 1:100), GATA3 (Abcam, ab110093; KT77; 1:500), KI67 (Abcam, ab15580; 1:500), AE13 (Abcam, ab16113; 1:300), AE15 (Santa Cruz Biotechnology, sc-80607; 1:300), p-Akt (Ser^473^) (Cell Signaling Technology, 9271S; 1:1000), p4E-BP1 (Ser^65^) (Cell Signaling Technology, 9451S; 1:1000), KRT71 (OriGene, BP5096; 1:1000) KRT75 (OriGene, BP5100; 1:1000), and RPL26 (Abcam, ab59567; 1:500 on paraffin sections using antigen retrieval and proteinase K treatment)

### Proteasome inhibition

HEK293 cells were treated with MG132 (MedChemExpress, no. HY-13259) for 24 hours at 10 μM before collection and analysis.

### Plasmids for transfections and coimmunoprecipitations

Plasmids used for transfections and coimmunoprecipitations were 3xFlag-4E-BP1_WT (pCW57.1), 3xFlag-4E-BP1_R62K (pCW57.1), HA-PADI4 (pCW57.1), eIF4E_6xHis (pUC.19), and LEF1_6xHis (pUC.19).

### Western blot and immunoprecipitation

HEK293FT or primary epidermal progenitor cells were transfected at the confluency of 80 to 90% in a 6-cm dish. Each plasmid (3 μg) was used for transfection using PEI (Sigma-Aldrich, no. 408727; 1:5). When transfecting inducible constructs, doxycycline was added after transfection (1 μg/ml). After 48 hours, cells were collected in 600 μl of radioimmunoprecipitation assay intraperitoneal buffer containing protease inhibitors (Pierce IP Lysis Buffer; Thermo Fisher Scientific, no. 87787). Cells were then lyzed on ice for 30 min and centrifuged at 13,000 rpm for 15 min at 4°C to pellet debris. The supernatant (100 μl) was saved as input control, and the other 500 μl were collected for immunoprecipitation assay. Immunoprecipitation was performed following the manufacturer’s recommendation. In short, for each reaction, 25 μl of HA-tagged beads (HA-Tag magnetic IP/Co-IP kit; Thermo Fisher Scientific, no. 13484229) or 50 μl of Flag-tagged beads (ANTI-DYKDDDDK MAG AGAROSE 1ML SETTLE; Thermo Fisher Scientific, no. 15875833) were used for immunoprecipitating HA-PADI4 or Flag-4E-BP1, respectively. Beads were collected using a magnetic stand and washed twice with lysis/wash buffer with protease inhibitor and, lastly, with ultrapure water. A “beads-only” negative control was included to exclude unspecific detection of light and heavy immunoglobulin G (IgG) chain. Boiling at 95°C for 5 min in 100 μl of nonreducing 5× Lane Marker Sample Buffer was used to release proteins (but prevent release of HA-antibody fragments) from the beads and allow for Western blot analysis. For reducing-gel analysis, 2.5 μl of 2 M dithiothreitol (DTT) was added. Western blot samples were run on an SDS–polyacrylamide gel electrophoresis (PAGE) gel (Bio-Rad) and transferred onto a polyvinylidene difluoride (PVDF) membrane (Merck Millipore). Membranes were blocked with 5% milk in tris-buffered saline (TBS) containing 0.1% Tween 20 for 1 hour at room temperature and incubated with primary antibody at 4°C overnight, followed by 1 hour of incubation with HRP-conjugated secondary antibody at room temperature. Blots were developed using SuperSignal West Femto Maximum Sensitivity Substrate (Thermo Fisher Scientific) or Immobilon Crescendo Western HRP substrate (Millipore, Merck) and imaged with ChemiDoc (Bio-Rad). The input sample represented 20% starting material used for immunoprecipitation.

Antibodies used for Western blot: anti-HA (Sigma-Aldrich, 11867423001; 1:5000), anti-Flag (Sigma-Aldrich, M2-F1804; 1:5000), anti-His (Abcam, ab9108; 1:5000), Akt (Cell Signaling Technology, 9272S; 1:1000), p-Akt (Ser^473^) (Cell Signaling Technology, 9271S; 1:1000), 4E-BP1 (Cell Signaling Technology, 9452S; 1:1000), p4E-BP1(Ser^65^) (Cell Signaling Technology, 9451S; 1:1000), 4E-BP1 (Thr^70^) (Cell Signaling Technology, 9455; 1:1000), S6 (Cell Signaling Technology, 2217S; 1:1000), pS6 (Ser^240/244^) (Cell Signaling Technology, 2215S; 1:1000), mTOR (Cell Signaling Technology, 2972S; 1:1000), p-mTOR (S2448) (Cell Signaling Technology, 2971S; 1:1000), eIF4E (Cell Signaling Technology, 9742S; 1:1000), peIF4E (S209) (Cell Signaling Technology, 9741S; 1:1000), Eif2a (Cell Signaling Technology, 9722S; 1:1000), pEif2a (S51) (Cell Signaling Technology, 9721S; 1:1000), LEF1 (Cell Signaling Technology, 2230T; clone C12A5; 1:1000), β-actin (Sigma-Aldrich, AC-74; 1:1000), β3-tubulin (Abcam, ab18207; 1:1000), anti–glyceraldehyde-3-phosphate dehydrogenase (Santa Cruz Biotechnology, 0411; 1:1000), puromycin/OPP (Merck, MABE342; clone 4G11; 1:500), α-Tubulin (Abcam, ab7291; DM1A; 1:1000), and pP70SK T389 (Cell Signaling Technology, 9205S). Secondary antibodies used were HRP-conjugated goat anti-rabbit, goat anti-chicken, donkey anti-rat, and donkey anti-mouse at 1:10,000 (Jackson ImmunoResearch)

### Detection of citrullinated residues using AMC kit

Seventy-two hours posttransfection, HEK293 cells were treated with 5 mM CaCl_2_ (Sigma-Aldrich, in dH_2_O) and 5 mM ionomycin [Thermo Fisher Scientific (Invitrogen), in DMSO] or DMSO for control, for 3 hours after which the cells were collected and processed for Western blot as described above. The Anti-Modified Citrulline Detection Kit (Merck Millipore, no. 17-347B) was used to assess the presence of citrullinated proteins according to the manufacturer’s instructions. In brief, the PVDF membrane was incubated with a 1:1 mixture of reagent A (0.025% FeCl_3_, 25% H_2_SO_4_, and 17% H_3_PO_4_) and reagent B (0.5% 2,3-butanedione monoxime, 0.25% antipyrine, and 0.5 M acetic acid) overnight at 37°C without agitation in an airtight, lightproof container. The membrane was then rinsed six times with water and blocked with 5% milk in TBS containing 0.1% Tween 20 for 1 hour at room temperature, incubated overnight with the primary anti-modified citrulline antibody (Merck Millipore, no. 17-347B), and diluted in blocking solution (TBS, 0.1% Tween 20, and 5% milk), at 4°C, followed by 1 hour of incubation with the anti-human IgG HRP-conjugated secondary antibody (also in blocking solution) at room temperature. Blots were developed using SuperSignal-Aldrich West Femto Maximum Sensitivity Substrate (Thermo Fisher Scientific, no. 34095) and imaged with ChemiDoc (Bio-Rad).

### 5-EU labeling and capturing of nascent rRNA

Cells were labeled with 0.2 mM 5-EU for 3 hours and collected in TRIzol LS Reagent for total RNA extraction. The 5-EU RNA was subsequently biotinylated, extracted using magnetic beads, and precipitated, all according to the manufacturer’s instruction. In brief, 5-EU RNA was conjugated with Biotin azide by click-chemistry. RNA was precipitated from the click reaction, and biotinylated 5-EU RNA was bound to Dynabeads MyOne Streptavidin T1 magnetic beads to separate the labeled 5-EU RNA from total RNA. Subsequently, after cDNA synthesis, 5-EU–labeled rRNA and total rRNA were analyzed by reverse transcription (RT)–qPCR (Thermo Fisher Scientific, no. C10365).

### Reverse transcription quantitative polymerase chain reaction

RNA was extracted from cells collected in TRIzol LS Reagent (Thermo Fisher Scientific) using a chloroform/RNAeasy kit (QIAGEN) hybrid protocol. In brief, chloroform was added to the TRIzol/cell solution in a 1:5 ratio (i.e., 200 μl of chloroform per milliliter of TRIzol) and vigorously mixed for 20 s. The samples were then allowed to settle for 3 min before being centrifuged at 10,000*g* for 18 min at 4°C. This allowed for the separation of phases, where the top, aqueous, phase contained the RNA and was carefully transferred into a clean microcentrifuge tube (Eppendorf). Slowly, an equal volume of 100% ethanol was added to the aqueous phase and mixed as needed. The total volume of the sample was loaded onto an RNeasy kit column (QIAGEN) and RNA extraction proceeded according to the manufacturer’s instructions. Concentrations and quality were assessed with NanoDrop 2000c (Thermo Fisher Scientific), and 100 ng of total RNA was used for cDNA synthesis using SuperScript IV Vilo (Thermo Fisher Scientific). RT-qPCR was run with selected primer pairs and SYBR Green on a ViiA 7 device or a 7500 fast system (both Applied Biosystems). Hypoxanthine-guanine phosphoribosyltransferase was used as an internal control.

### Luciferase reporter assay

PADI4 cKO, OE, and WT primary epidermal progenitor cells were transfected with Tagged-Cap, Tagged-IRES (Addgene, nos. 35572 and 35570, respectively) (*50*), and Renilla-luciferin 2-monooxygenase RLuc transfection baseline control (in a ratio of 1:10) using Lipofectamine LTX with PLUS reagent (Thermo Fisher Scientific, no. 15338100) according to manufacturer’s instructions in a 24-well format. Cells were harvested 48 hours posttransfection and analyzed for luciferase activity and RLuc using the DualGlo luciferase assay system (Promega, no. E2920) on a GloMAX reader.

### Recombinant protein production

Recombinant PADI4, 4E-BP1WT, and 4EBP1R62K were expressed and purified with the help of the Protein Science Core Facility at the Karolinska Institutet according to the established standardized protocols. In short, both PADI4 and 4E-BP1 constructs were N-terminally tagged with His-Spidersilk-3C and transformed into *Escherichia coli* BL21 (DE3) T1R pRARE2 cells. The cells were cultivated in Terrific Broth medium. Protein expression was induced with isopropyl-β-d-thiogalactopyranoside, and proteins were purified by immobilized metal-ion chromatography, followed by size exclusion chromatography. The purification batches were aliquoted, flash-frozen in liquid nitrogen, and stored at −80°C. The yield was 20 to 28 mg per protein. The batch storage buffer contained 20 mM Hepes, 300 mM NaCl, 10% glycerol, and 2 mM Tris(2-carboxyethyl)phosphine (TCEP; pH 7.5). Batch purity was analyzed using SDS-PAGE. For the SDS-PAGE, ~4 μg of protein was loaded. Protein concentration was calculated from ultraviolet (UV) absorbance at 280 nm using a theoretical extinction coefficient. For the following protein sequences, bolding indicates the mutated site.

#### Purified 4E-BP1 WT protein sequence

MSDKIIHLTDDSFDTDVLKADGAILVDFWAEWCGPCKMIAPILDEIADEYQGKLTVAKLNIDQNPGTAPKYGIRGIPTLLLFKNGEVAATKVGALSKGQLKEFLDANLALEVLFQGHMHHHHHHSSGVDLGTENLYFQSMGSAGSSCSQTPSRAIPTRRVALGDGVQLPPGDYSTTPGGTLFSTTPGGTRIIYDRKFLMEC**R**NSPVAKTPPKDLPAIPGVTSPTSDEPPMQASQSQLPSSPEDKRAGGEESQFEMDI*

#### Purified 4E-BP1 R62K protein sequence

MSDKIIHLTDDSFDTDVLKADGAILVDFWAEWCGPCKMIAPILDEIADEYQGKLTVAKLNIDQNPGTAPKYGIRGIPTLLLFKNGEVAATKVGALSKGQLKEFLDANLALEVLFQGHMHHHHHHSSGVDLGTENLYFQSMGSAGSSCSQTPSRAIPTRRVALGDGVQLPPGDYSTTPGGTLFSTTPGGTRIIYDRKFLMEC**K**NSPVAKTPPKDLPAIPGVTSPTSDEPPMQASQSQLPSSPEDKRAGGEESQFEMDI*

#### Purified PADI4 protein sequence

MSDKIIHLTDDSFDTDVLKADGAILVDFWAEWCGPCKMIAPILDEIADEYQGKLTVAKLNIDQNPGTAPKYGIRGIPTLLLFKNGEVAATKVGALSKGQLKEFLDANLALEVLFQGHMHHHHHHSSGVDLGTENLYFQSMAQGAVIHVAPEQPTHAVCVVGTATPLDVRGSAPKGYTTFGITASPGVIVDVIHGPPVKKSTMGASKWPLDPELEVTLQVKAASSRTDDEKVRVSYYGPKTSPVQALIYITGVELSLSADVTRTGRVKPAQAGKDQSTWTWGPGGRGAILLVNCDKEDPQASGMDFEDDKILDNKDLQDMSPMTLSTKTPKDFFEKYQLVLEVPKAKMNRVRVFRATRGKLPSRYKVALGPQQFSYCLELPGGQHSTDFYVEGLAFPDADFKGLIPLTISLLDKSNPELPEALVFQDSVTFRVAPWIMTPNTQPPQEVYVCRVSDNEDFLKSLATLTKKAKCKLTVCPEEENIDDQWMQDEMEIGYIQAPHKTLPVVFDSPRDRGLKDFPVKRVMGPNFGYVTRKLYMSELTGLDAFGNLEVSPPVTVRGKEYPLGRILIGNSGYSSSESRDMHQALQDFLSAQQVQAPVRLFSDWLFVGHVDEFLSFVPARDKQGFRLLLSSPRACYQLFQELQSQGHGEATLFEGLKRKRQTINEILSNKKLRDQNAYVESCIDWNRAVLKRELGLAEGDIIDIPQLFKLAGNSRGNSKAQAFFPNMVNMLVLGKYLGIPKPFGPIIDGHCCLEEEVRSHLEPLGLHCTFINDFYTYHVYNGEVHCGTNVRRKPFTFKWWHMVP*

### In vitro citrullination assay

In vitro citrullination was carried out with recombinant PADI4 in deimination buffer [50 mM Hepes (pH 7.5), 10 mM CaCl_2_, and 4 mM DTT] with recombinant 4E-BP1 WT and 4E-BP1 R62K mutant at 37°C for 1 hour. Recombinant histone H3 (Abcam, no. ab198757) was used as a positive control. The 4E-BP1 proteins (2 μg) and histone H3 (1 μg) were used for the reaction. Samples were dissolved in sample Laemmli buffer, and 4E-BP1 samples were run on 8% SDS-PAGE gels and histone H3 on 12% SDS-PAGE for immunoblot analysis using an anti-citrulline antibody (Merck, no. 17-347B) (*25*, *65*, *66*).

### Mass spectrometry sample preparation

Cell pellets were solubilized with 50 μl of 8 M urea/100 mM NaCl and 10 μl of 1% ProteaseMAX in 100 mM AmBic (pH 8.5) following sonication in water bath for 10 min. Following 40 μl of 50 mM AmBic was added together with 1 μl of 100× protease inhibitor cocktail (Roche) before probe sonicated with VibraCell probe (Sonics & Materials Inc.) for 40 s, with pulse of 2/2, at 20% amplitude. Protein concentration was measured by bicinchoninic acid (BCA) assay (Thermo Fisher Scientific). An aliquot of 25 μg samples was digested with addition of 0.5 μg of sequencing grade modified trypsin (Promega) and incubated for 16 hours at 37°C. The digestion was stopped with 5 μl of cc. formic acid, incubating the solutions at room temperature for 5 min. The sample was cleaned on a C18 Hypersep plate with 40-μl bed volume (Thermo Fisher Scientific) and dried using a vacuum concentrator (Eppendorf). Biological samples were labeled with TMT-10plex reagents in random order adding 100 μg of TMT-reagent in 30 μl of dry acetonitrile (ACN) to each digested sample resolubilized in 70 μL of 50 mM triethylammonium bicarbonate (TEAB) and incubating at room temperature for 2 hours. The labeling reaction was stopped by adding 11 μl of 5% hydroxylamine and incubated at room temperature for 15 min before combining them in one vial. An aliquot of this analytical sample (22 μg) was cleaned on StageTip C18 (Thermo Fisher Scientific) and dried before resuspended in 15 μl of 0.1% formic acid and 2% ACN. The sample with combined TMT-labeled biological replicates was fractionated by high-pH reversed-phase after dissolving in 50 μl of 20 mM ammonium hydroxide and was loaded onto an Acquity bridged ethyl hybrid C18 UPLC column (2.1 mm inner diameter by 150 mm, 1.7-μm particle size, Waters) and profiled with a linear gradient of 5 to 60% 20 mM ammonium hydroxide in ACN (pH 10.0) over 48 min, at a flow rate of 200 μl/min. The chromatographic performance was monitored with a UV detector (Ultimate 3000 UPLC, Thermo Scientific) at 214 nm. Fractions were collected at 30-s intervals into a 96-well plate and combined in 12 samples concatenating 8-8 fractions representing peak peptide elution.

### LC-MS/MS data acquisition

Peptides of TMT-labeled fractions were reconstituted in solvent A, and ~2 μg of samples were injected on a 50-cm-long EASY-Spray C18 column (Thermo Fisher Scientific) connected to an Ultimate 3000 nanoUPLC system (Thermo Fisher Scientific) using a 90 min long gradient: 4 to 26% of solvent B (98% acetonitrile and 0.1% formaldehyde) in 90 min, 26 to 95% in 5 min, and 95% of solvent B for 5 min at a flow rate of 300 nl/min. Mass spectra were acquired on a Orbitrap Fusion Lumos tribrid mass spectrometer (Thermo Fisher Scientific) ranging from mass/charge ratio (*m/z*) 375 to 1700 at a resolution of *R* = 120,000 (at *m/z* 200) targeting 8 × 10^5^ ions for maximum injection time of 54 ms, followed by data-dependent higher-energy collisional dissociation fragmentations of precursor ions with a charge state 2+ to 8+, using 45-s dynamic exclusion. The tandem mass spectra of the top precursor ions were acquired in 3-s cycle time with a resolution of *R* = 50,000, targeting 1 × 10^4^ ions for maximum injection time of 86 ms, setting quadrupole isolation width to 1.4 Th and normalized collision energy (NCE) to 35%.

Peptides of label-free samples were separated with the same parameters as above, and mass spectra were acquired on a Q Exactive HF hybrid quadrupole Orbitrap mass spectrometer (Thermo Fisher Scientific) in *m/z* 350 to 1600 at a resolution of *R* = 120,000 (at *m/z* 200), targeting 5 × 10^6^ ions for maximum injection time of 54 ms and collecting tandem mass spectra of top 18 precursors with 1.4 Th isolation width at 30,000 mass resolution applying 28% NCE.

### Mass spectrometry data analysis

Acquired raw data files were analyzed using Proteome Discoverer v2.4 (Thermo Fisher Scientific) with Mascot Server v2.5.1 (Matrix Science Ltd., UK) search engine against mouse protein database (Swiss-Prot). A maximum of two missed cleavage sites were allowed for full tryptic digestion while setting the precursor and the fragment ion mass tolerance to 10 parts per million and 0.02, respectively. Carbamidomethylation of cysteine was specified as a fixed modification, while TMT6plex on lysine and N-termini, oxidation on methionine, as well as deamidation of asparagine and glutamine (for citrullination also on arginine) were set as dynamic modifications. Initial search results were filtered with 5% false discovery rate (FDR) using Percolator node in Proteome Discoverer. Peptides where citrullination was located to the C terminus were identified and excluded from the final functional analysis. All remaining citrullinated peptides were included in the downstream functional analysis, without taking the overall protein expression level into account. No determination of citrullination stoichiometry on individual proteins was performed. It is, thus, possible that the reported changes in citrullination profiles reflect changes in the proteome rather than citrullinome. Peptide identification did not include detection of the neutral loss of isocyanic acid, a characteristic and specific marker for citrullinated proteins (*67*). Proteome quantification was based on the TMT-reporter ion intensities.

### Statistical and bioinformatic analyses

GraphPad Prism was used for statistical analyses, including the use of multiple unpaired *t* test and analysis of variance (ANOVA) when applicable. Error bars show the SD or standard error of the mean as specified in the figure legends. GO analysis (*68*) for protein enrichment was performed using the PANTHER classification system (*69*) (http://pantherdb.org/), and enrichment score was calculated by −log(*P* value) × log_2_(fold enrichment) (*32*). The STRING database (*70*) was used to generate protein maps of interactions of citrullinated proteins in PADI4 overexpressing cells. The *n* of in vivo quantifications is specified in the corresponding figure legend. In vitro experiments have been performed multiple times to verify the conclusions displayed in the representative figures.

## Supplementary Materials

### The PDF file includes:

Figs. S1 to S6 Legends for tables S1 to S4 Fig. S4K uncropped Western blots
