# Supplementary material for: The citrullinating enzyme PADI4 governs progenitor cell proliferation and translation in developing hair follicles
Source: Sci Adv. 2025 Sep 12;11(37):eadx4511. doi: 10.1126/sciadv.adx4511 (PMC12429026; doi:10.1126/sciadv.adx4511)
Supplement: Supplementary file 1 — Figs. S1 to S6 Legends for tables S1 to S4 Fig. S4K uncropped Western blots [file sciadv.adx4511_sm.pdf]

Science Advances
AAAS

Supplementary Materials for

The citrullinating enzyme PADI4 governs progenitor cell proliferation and translation in developing hair follicles

Kim Vikhe Patil *et al.*

Corresponding author: Maria Genander, maria.genander@ki.se

Sci. Adv. **11**, eadx4511 (2025)
DOI: 10.1126/sciadv.adx4511

The PDF file includes:

Figs. S1 to S6
Legends for tables S1 to S4
Fig. S4K uncropped Western blots

Other Supplementary Material for this manuscript includes the following:

Tables S1 to S4

Fig. S1. *Padi4* expression in the developing hair follicle. Related to Fig. 1.

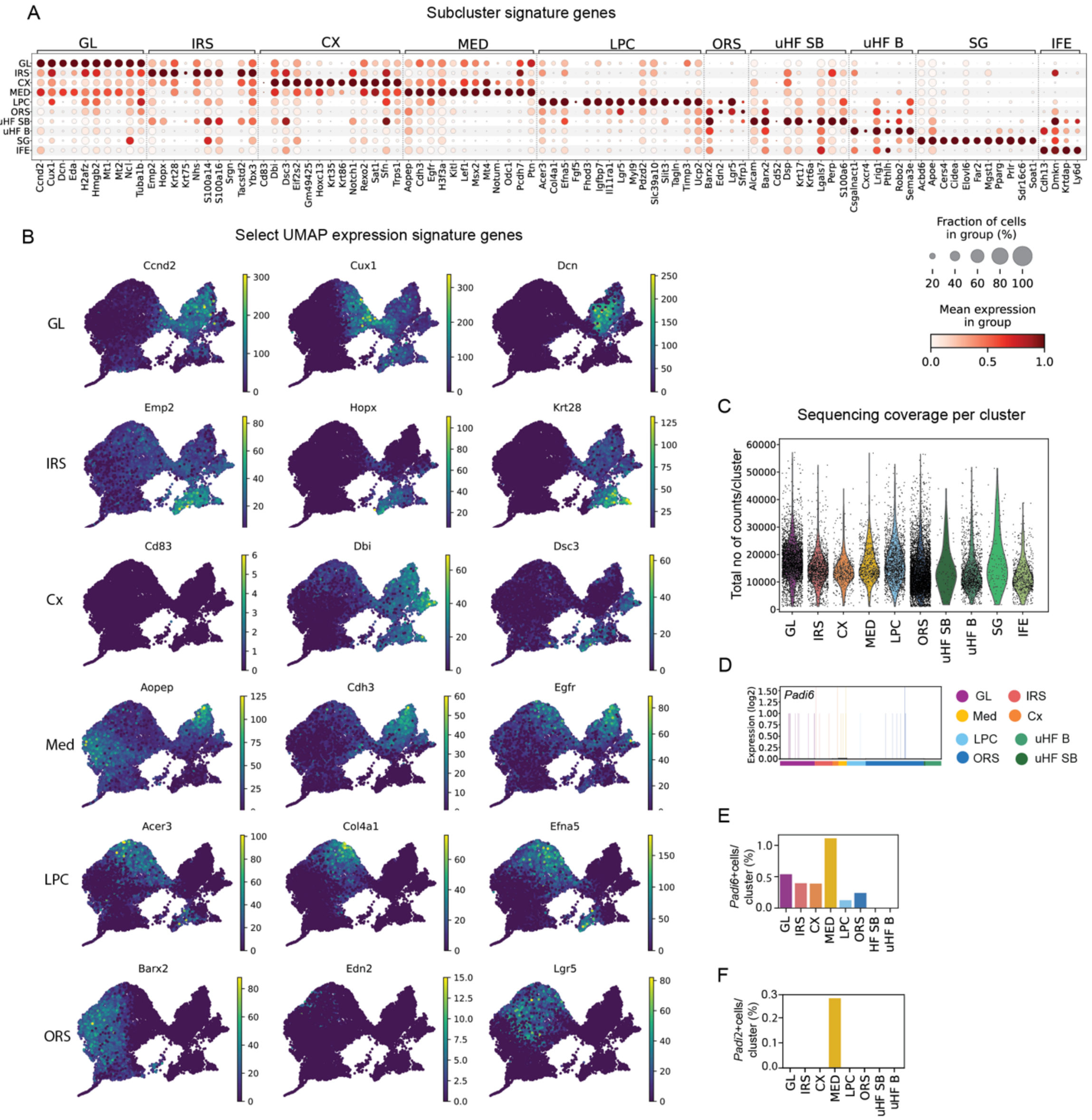

(**A**) Marker gene expression dot plot for main clusters. (**B**) UMAPs highlighting expression of top 3 signature genes in six main HF clusters. (**C**) Violin plot comparing the total number of single cell sequencing counts per HF cluster. (**D**) Expression plot displaying *Padi6* levels in HF clusters. (**E**) Frequency of *Padi6*-positive cells per HF cluster (percentage). (**F**) Frequency of *Padi2*-positive cells per HF cluster (percentage).

Fig. S2. PADI4 negatively regulates HF progenitor cell proliferation. Related to Fig. 2.

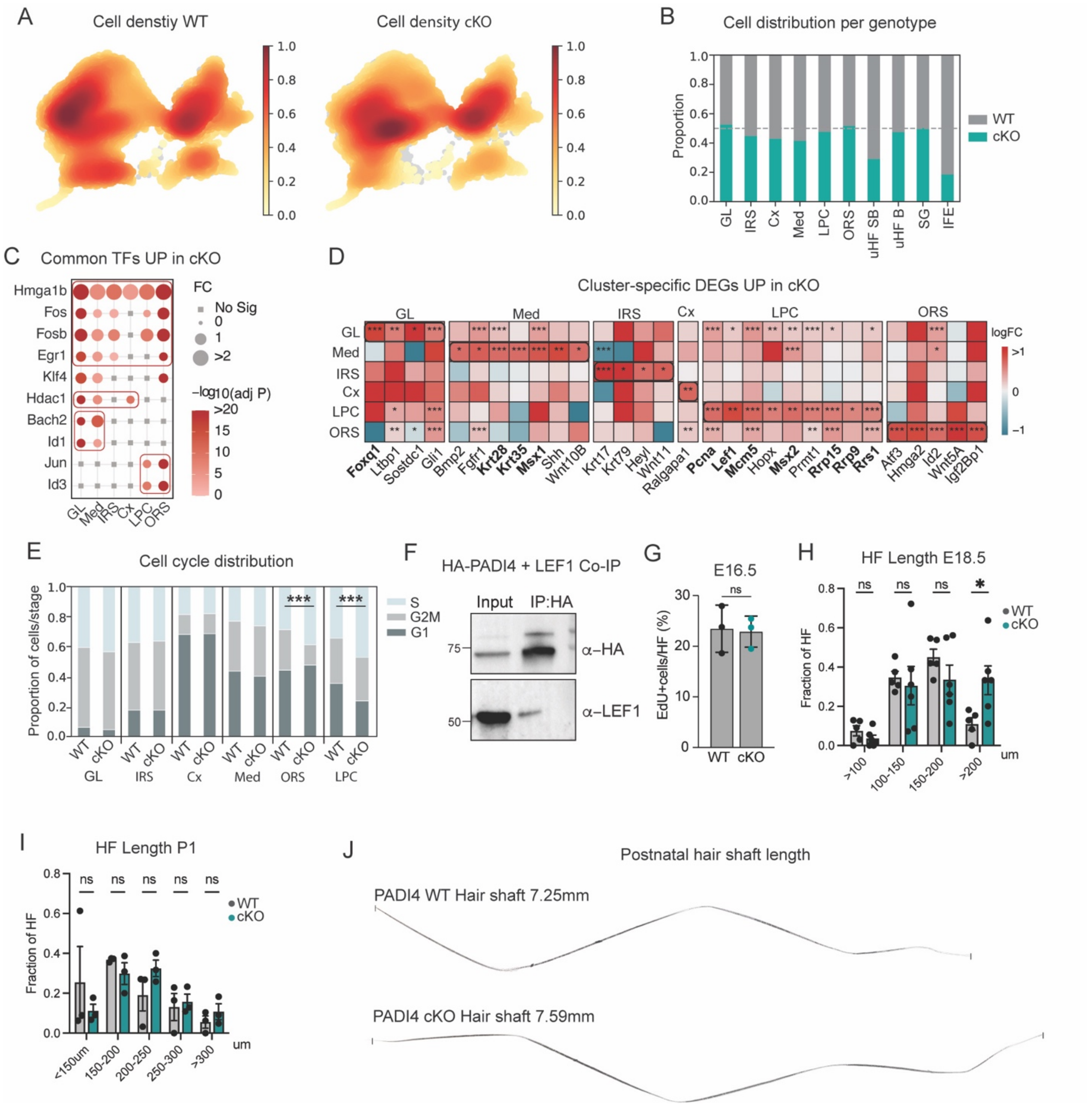

(**A**) Density plot displaying distribution of PADI4 WT and cKO HF cells. (**B**) Contribution of PADI4 WT or cKO cells to each cluster. The horizontal grey line indicates 50%. (**C**) Dot plot of transcriptional and chromatin regulators upregulated in several HF clusters in PADI4 cKO compared to WT. *Hmga1b* is upregulated in all six HF clusters. (**D**) Heat map highlighting cluster-specific genes in six main HF clusters. (**E**) Predicted distribution of cell cycle states comparing PADI4 WT and cKO HF clusters. (**F**) Co-immunoprecipitation of HA-PADI4 and LEF1-6xHis in transiently transfected HEK293 cells. (**G**) Quantification of proliferation using EdU in PADI4 WT and cKO HFs at E16.5. (**H**) Representation of HF length at E18.5 reveals an increase in the

percentage of long (>200 μm) HFs in the absence of PADI4. (**I**) Distribution of HF lengths at P1 reveal no difference when comparing PADI4 WT and cKO HFs. (**J**) Representative images of PADI4 WT and cKO hairs plucked from adult (8 weeks old) mice.

(**C-D**) DEGs were calculated using the Wilcoxon rank-sum method. A gene was considered differentially expressed if adjusted p-value <0.05 and log2 fold change >0.5. (**E**) Chi-square test of independence. (**G**) Two-sided unpaired student's t-test. (**H-I**) Two-way ANOVA. ns $p > 0.05$, * $p < 0.05$, ** $p < 0.05$, ***$p < 0.001$. Data are represented as mean±SD, n=3 or more for each condition, each dot represents one animal (**H-I**).

Fig. S3. Characterization of the PADI4-dependent citrullinome. Related to Fig. 4.

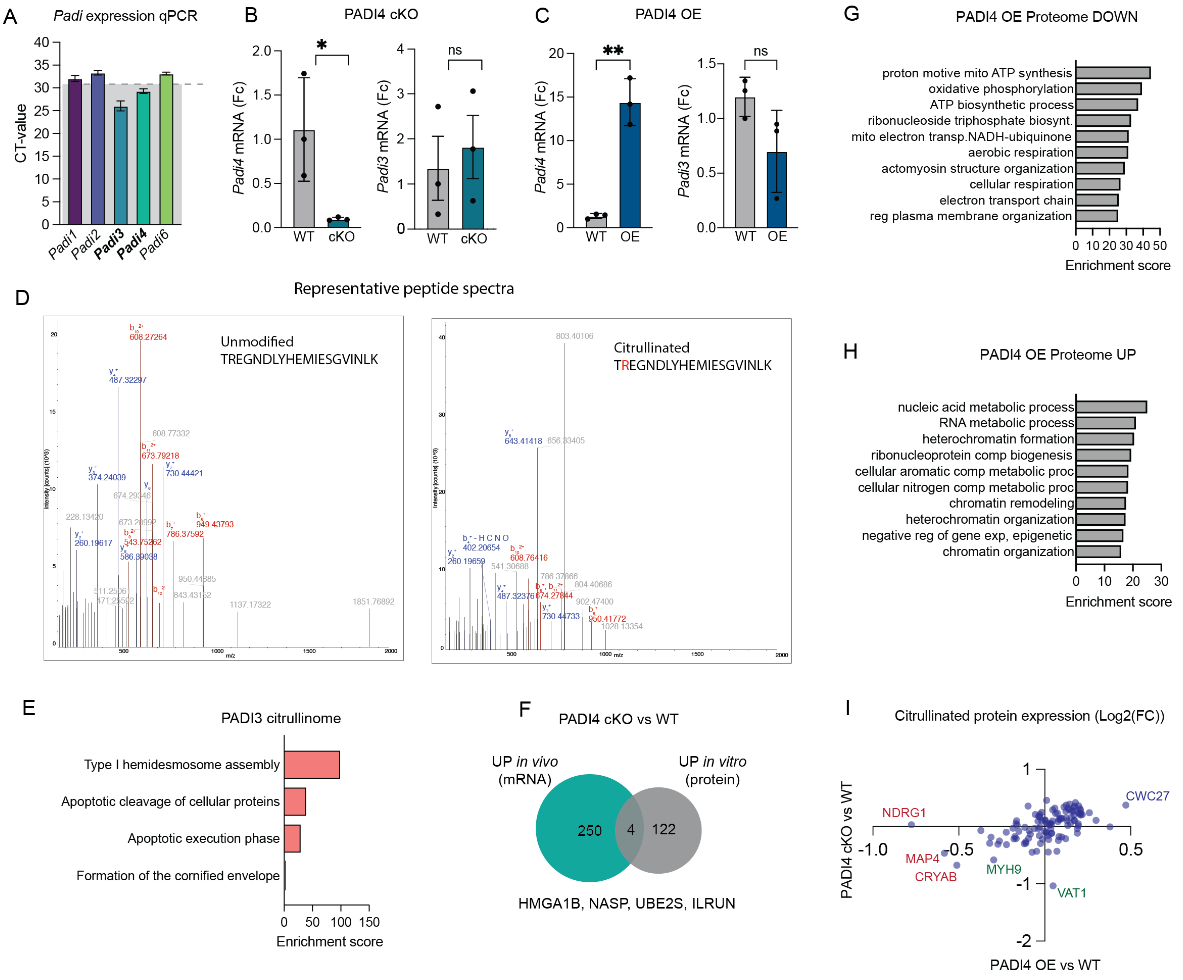

(**A**) CT-values for *Padi* enzyme families in WT primary progenitor cells. Only *Padi3* and *Padi4* are significantly expressed (CT-values higher than 32 is considered as no expression) n=3. (**B**) mRNA expression of *Padi4* and *Padi3* in primary progenitor cells comparing PADI4 WT and cKO. (**C**) mRNA expression of *Padi4* and *Padi3* in primary progenitor cells comparing PADI4 WT and OE. (**D**) Representative MS/MS spectra of unmodified and citrullinated peptide. (**E**) Gene Ontology analysis of citrullinated proteins found in PADI4 cKO progenitor cells (likely PADI3-mediated). (**F**) Venn diagram overlaying genes upregulated in the inner HF cell clusters (254) with proteins upregulated in mass spectrometry data (126) identified when comparing PADI4 WT to cKO. Only four targets are common (*Hmga1b*, *Nasp*, *Ube2s* and *Ilrun*). (**G**) Top 10 Gene Ontology categories associated with proteins down regulated in PADI4 OE compared to WT. (**H**) Top 10 Gene Ontology categories associated with proteins upregulated in PADI4 OE compared to WT. (**I**) Expression levels of citrullinated proteins in the presence (PADI4 OE) or absence (PADI4 cKO) of citrullination. Citrullination fails to target proteins for degradation.

Two-sided unpaired student's t-test (**B-C)** ns $p > 0.05$, * $p < 0.05$, ** $p < 0.01$. Data are represented as mean±SD, each dot represents one biological replicate (**B-C)**.

Fig. S4. PADI4 inhibits global translational output by affecting ribosomal biogenesis. Related to Fig. 5.

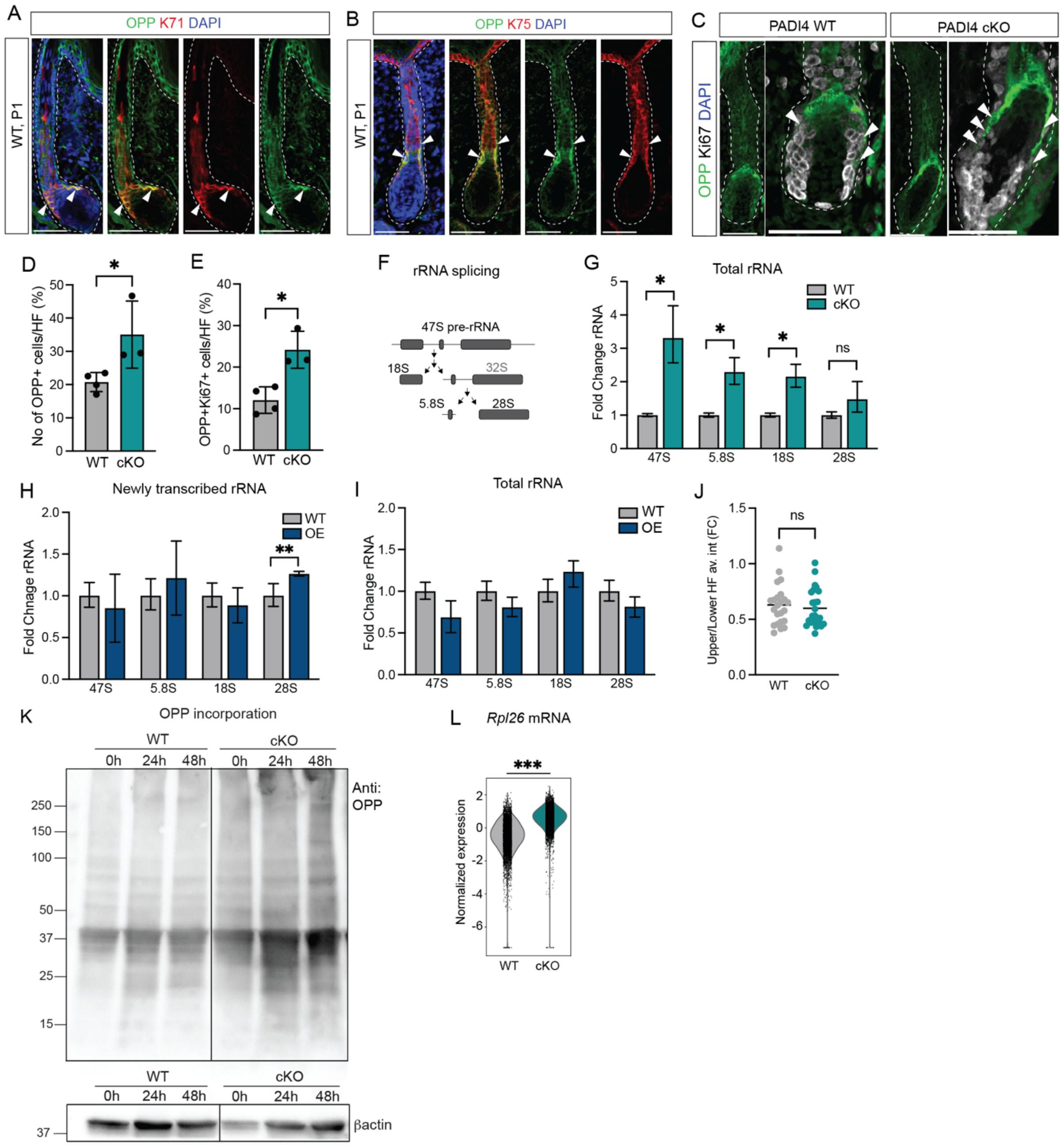

(**A-B**) Protein translation rates, as judged by OPP incorporation (green) are highest in differentiated K71+ IRS (**A**) and K75+ HS (**B**) lineages. (**C**) Labelling of OPP- and Ki67-expressing cells in PADI4 WT and cKO HFs at E18.5. (**D**) Quantification of OPP-positive cells in PADI4 WT and cKO HFs at E18.5. (**E**) Quantification of OPP+Ki67+ cells per HF comparing

PADI4 WT and cKO at E18.5. (**F**) Illustration of rRNA splicing. rRNA species are required for a functional ribosome. (**G**) Total rRNA expression of specific rRNA species (47S, 5.8S, 18S, and 28S) in PADI4 WT compared to cKO progenitor cells. Normalized to PADI4 WT expression. (**H**) Quantification of nascent rRNA transcription (3h EU incorporation) in PADI4 WT and OE progenitor cells. (**I**) Total rRNA expression of specific rRNA species (47S, 5.8S, 18S, and 28S) in PADI4 WT compared to OE progenitor cells. Normalized to PADI4 WT expression. (**J**) The upper/lower ratio of 47S signal intensity in PADI4 WT and cKO HFs is not different, confirming an overall higher rRNA synthesis in the absence of PADI4. (**K**) OPP incorporation in PADI4 WT and cKO progenitor (0h) and differentiated (24h and 48h) cells. PADI4 cKO progenitor cells have higher base line OPP incorporation, which is maintained during differentiation. ß-actin is used as a loading control. (**L**) Violin plot or normalized *Rpl26* expression in P1 WT and PADI4 cKO HF cells. Log2 fold change between WT and cKO expression is log2FC=0.95 with a significance of ***adjusted p-value<0.001 as determined by a Wilcoxon test.

(**D-E, G-J**) Two-sided unpaired student's t-test. **(L)** Wilcoxon test. ns $p > 0.05$, * $p < 0.05$, ** $p < 0.01$, *** $p < 0.001$. Scale bars: 50µm (**A**-**C**). Data are represented as mean±SD, each dot represents one animal (**D-E**) or one biological replicate (**G-I**) or one HF (**J**).

Fig. S5. PADI4 rewires the translational machinery in progenitor cells. Related to Fig. 6.

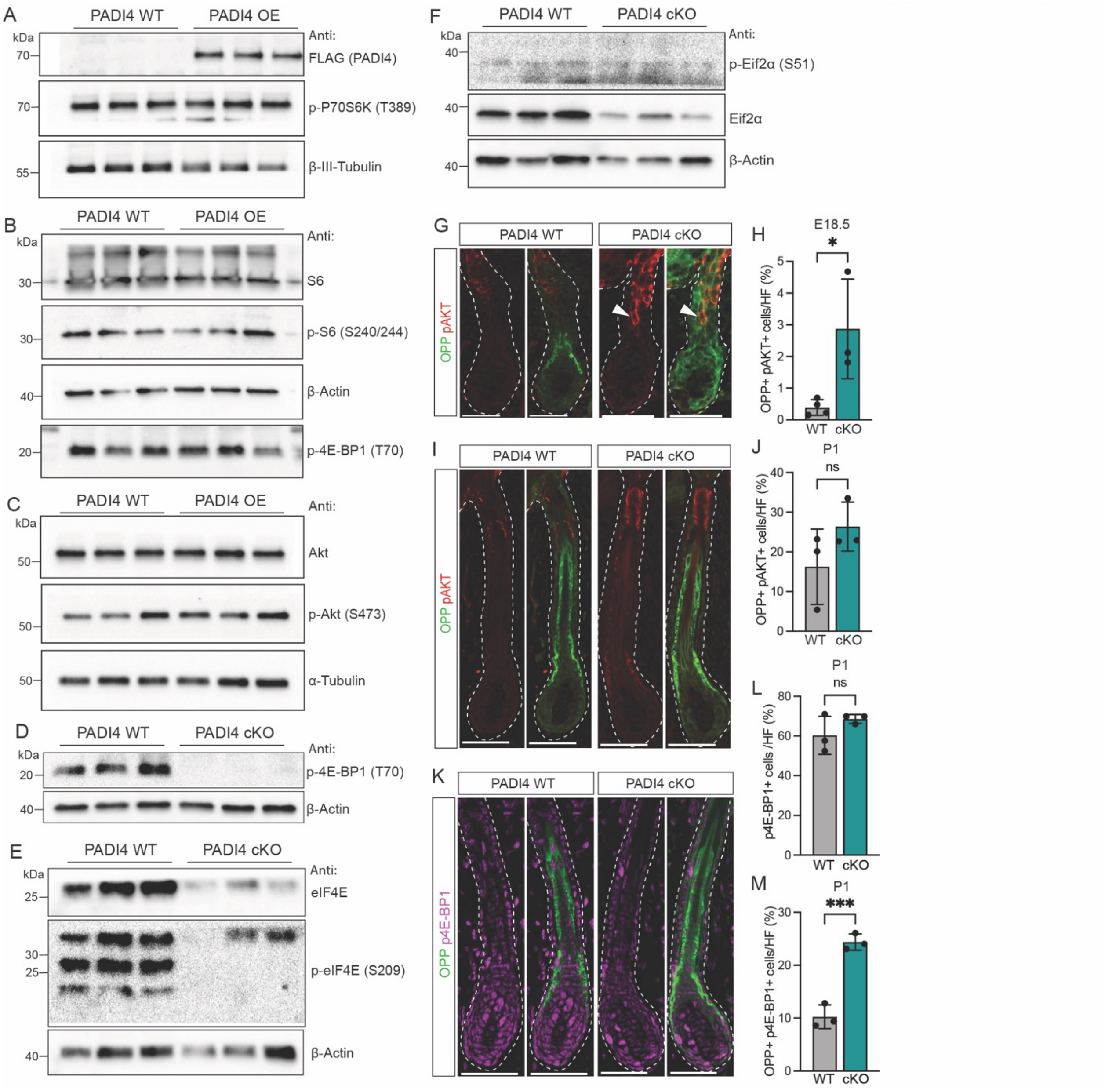

(**A**) Flag-tagged PADI4 overexpression is verified by blotting for FLAG. PADI4 OE does not change the levels of phosphorylated P70S6K (T389). (**B**) PADI4 OE does not lead to changes in overall S6, pS6 (S240/244) or p4E-BP1 (T70) levels. (**C**) AKT and pAKT (S473) are not changed when comparing PADI4 WT and OE progenitor cells. (**D**) Thr70 phosphorylation levels of 4E-BP1 is reduced in the absence of PADI4. (**E**) Protein levels of eIF4E in PADI4 WT and cKO progenitor cells. (**F**) Eif2a activity and level is reduced in the absence of PADI4. β-III-Tubulin, α-Tubulin, and β-Actin were used as loading controls (**A-F**). (**G**) pAKT is prominent in the upper, differentiated, part of the HF at E18.5. (**H**) Quantification of the number of pAKT/OPP double

positive cells comparing PADI4 WT and cKO HFs at E18.5. (**I**) pAKT is prominent in the upper, differentiated, part of the HF at P1. (**J**) Quantification of the number of pAKT/OPP double positive cells comparing PADI4 WT and cKO HFs at P1. (**K**) Localization of p4E-BP1 and OPP in PADI4 WT and cKO HFs at P1. (**L**) Quantification of percentage of p4E-BP1 expressing cells in the HF comparing PADI4 WT and cKO. (**M**) Quantification of OPP/p4E-BP1 double positive cells at P1.

Two-sided unpaired student's t-test (**H, J, L** and **M)** ns $p > 0.05$, * $p < 0.05$, ** $p < 0.01$, *** $p < 0.001$. Scale bars: 50µm (**G**, **I** and **K**). Data are represented as mean±SD, each dot represents one animal (**H, J, L and M)**.

Fig. S6. PADI4 binds and primes 4E-BP1 for phosphorylation. Related to Fig. 7.

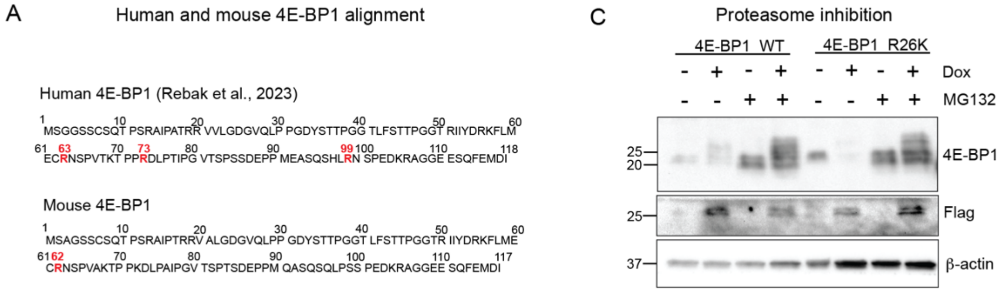

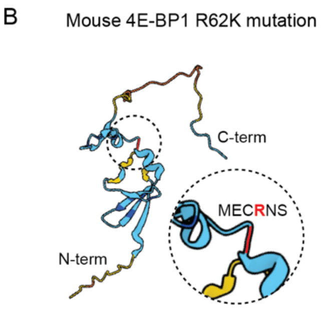

D

Protein	Epidermal Progenitors This work	Human Neutrophils Rebak., PMID 38321148	Phosphorylation
AKT1	Not Cit	R48	S473
mTOR (TORC1)	Not Cit	Not Cit	S2448
4E-BP1	Not Cit* (R62)	R63, R73, R99	S65
S6 (PPS6)	Not Cit	R22, R25, R31, R72, R74, R87, R88, R92, R98, R131, R132, R154, R159, R178, R183, R189, R190, R191, R231, R232, R233, R238	S235, S236, S240, S244
p70S6K (RPS6KB1)	Not Cit	R81, R321	T389
eiF2alpha (EIF2S1)	R173	R7, R113, R173, R292, R295	S51
eiF3B	R165	Not Cit	S154
eiF3I	R229	R20, R77	N.D.

*Not citrullinated in mass spectrometry data, verified in vitro (Fig. 7)

(**A**) Amino acid alignment of human and mouse 4E-BP1. Human arginine residues R63, R73 and R97, reported to be citrullinated (13), are marked in red. Only R63 is conserved to mouse 4E-BP1 (mouse R62). **(B)** AlphaFold generated protein structure of mouse 4E-BP1 highlighting R62K mutation (red). (**C**) Flag-tagged 4E-BP1_WT or 4E-BP1_R62K overexpression using doxycycline in the presence or absence of the proteasomal inhibitor MG132. Both 4E-BP1_WT and R62K accumulate with similar dynamics, indicating that the mutated 4E-BP1 protein behaves largely normal. (**D**) Table outlining citrullination and phosphorylation residues of translational mediators probed in Fig. 6 comparing data from this work to Rebak et al (13). S6 is citrullinated in 22 arginine residues, some of which are in proximity to C-terminal phosphorylation sites.

Supplemental table legends

Table S1. Differentially expressed genes (DEGs) defining each HF cluster.
Table S2. Differentially expressed genes (DEGs) per HF cluster, comparing PADI4 WT and cKO HFs.
Table S3. Mass spectrometry data from PADI4 WT, cKO and OE epidermal progenitor cells, including the proteome as well as the identified citrullinated peptides.
Table S4. Comparison of proteins and genes upregulated in PADI4 cKO vs WT progenitor cells (proteins) or HFs (genes).

Uncropped Western Blots

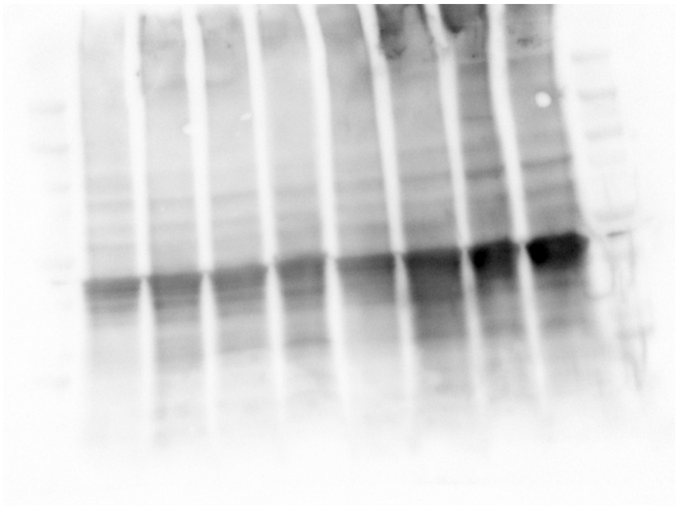

From Figure S4K: OPP incorporation, including 0h, 24h, 48h and 72h (not included in S4K, lane 4 and 8), comparing PADI4 WT and cKO epidermal progenitor cells.

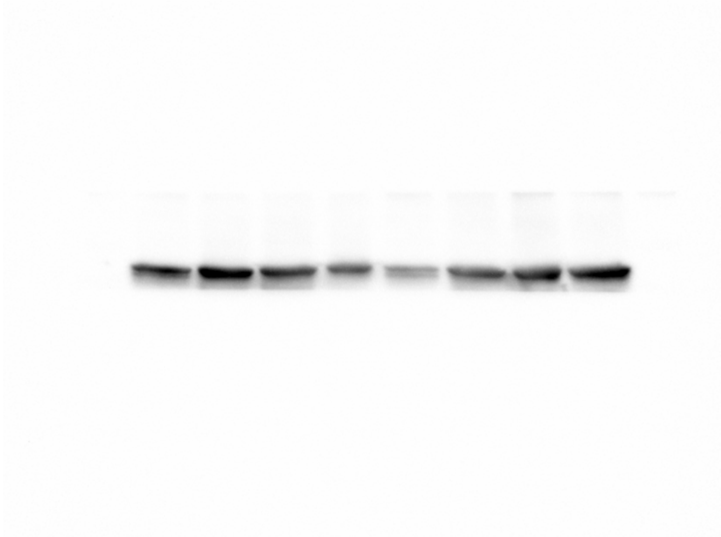

From Figure S4K: Actin loading control, including 0h, 24h, 48h and 72h (not included in S4K, lane 4 and 8), comparing PADI4 WT and cKO epidermal progenitor cells.
